# Conservative Oxygen Targets in Mechanically Ventilated Patients (OXY-BREATHES): A Systematic Review and Meta-Analysis of Randomized Controlled Trials

**DOI:** 10.1097/CCM.0000000000007031

**Published:** 2026-02-09

**Authors:** Nhan Nguyen, Nghi Bao Tran, Nathalia Alves de Barros e Lyra, Yacin Zawam, David Downes, Vinh Quang Tri Ho, Vy Ngoc Dan Nguyen, Ha Duc Thien Le, Jafar Aljazeeri

**Affiliations:** 1 Faculty of Medicine, University of Debrecen, Debrecen, Hungary.; 2 Faculdade Pernambucana de Saude, Recife, Brazil.; 3 Southern Ohio Medical Center, Portsmouth, OH.; 4 Department of Rural Medicine, The University of New England, Armidale, NSW, Australia.; 5 Department of Nursing, The University of Melbourne, Melbourne, VIC, Australia.; 6 University of Pittsburgh Medical Center, Pittsburgh, PA.; 7 Drexel University College of Medicine, Philadelphia, PA.; 8 Medical City Hospital for Military and Security Services, Muscat, Oman.

**Keywords:** cardiac arrest, conservative oxygen targets, mechanical ventilation, mortality, sepsis, ventilator-free days

## Abstract

**OBJECTIVES:**

To evaluate the efficacy and safety of conservative (oxygen saturation [Spo_2_] 88–94% or Pao_2_ < 80 mm Hg) vs. liberal oxygen targets (Spo_2_ ≥ 94% or Pao_2_ ≥ 90 mm Hg) in mechanically ventilated critically ill adults.

**DATA SOURCES:**

PubMed, Cochrane CENTRAL, Embase, and ClinicalTrials.gov.

**STUDY SELECTION:**

We conducted the OXY-BREATHES, a systematic review and meta-analysis of randomized controlled trials (RCTs) comparing conservative vs. liberal oxygen targets in mechanically ventilated ICU patients. Primary outcomes were 90-day mortality and ICU length of stay. Secondary outcomes included ventilator- and vasopressor-free days, renal replacement therapy, nosocomial pneumonia, and cardiac or cerebral ischemic events. Subgroup analyses included patients with sepsis/septic shock and post-cardiac arrest.

**DATA EXTRACTION:**

Data were collected according to study selection criteria. Certainty of evidence was appraised with Grading of Recommendations, Assessment, Development, and Evaluation, and risk of bias with the Cochrane tool. Data were analyzed using a random-effects model.

**DATA SYNTHESIS:**

Nine RCTs enrolling 20,447 patients were included. Conservative and liberal targets showed no substantial differences in 90-day (risk ratio [RR], 1.01; 95% CI, 0.94–1.09) or ICU length of stay (mean difference [MD], –0.17; 95% CI, –0.41 to 0.06). Secondary outcomes, including organ support-free days and the incidence of adverse events, were comparable between groups. In subgroup analyses, conservative targets yielded more vasopressor-free days in septic patients (MD, 2.0; *p* = 0.008) and a potential survival benefit in post-cardiac arrest patients (RR, 0.89; *p* = 0.05). Certainty of evidence was rated moderate for 90-day mortality, ICU length of stay, vasopressor-free days, and ventilator-free days; low for renal replacement therapy and nosocomial pneumonia; and very low for cerebral and cardiac ischemia due to imprecision and open-label trial designs.

**CONCLUSIONS:**

Conservative oxygenation is comparable to liberal oxygen targets in mechanically ventilated critically ill patients, with possible advantages in sepsis and post-cardiac arrest. Future condition-specific RCTs are warranted to define optimal ICU oxygen strategies.

**KEY POINTS**

**Question**: Does conservative oxygenation provide better outcomes for mechanically ventilated patients?

**Finding**: Overall, conservative oxygen targets yielded outcomes comparable to liberal targets. However, subgroup analyses showed that conservative oxygenation was associated with more vasopressor-free days in patients with sepsis and suggested a potential survival benefit in post-cardiac arrest patients.

**Meaning**: Oxygen therapy should be individualized in mechanically ventilated patients, as optimal targets vary across clinical subgroups. Conservative oxygenation (oxygen saturation, 88–94%) may benefit patients with sepsis and post-cardiac arrest, while caution is needed in acute respiratory distress syndrome. Broad trial inclusion criteria introduce heterogeneity, highlighting the need for condition-specific randomized controlled trials.

In the ICU, oxygen therapy is essential for preventing severe hypoxemia, which can precipitate multiple organ failure and exacerbate systemic inflammation. Critically ill patients often require varying levels of respiratory support, particularly invasive mechanical ventilation (1). However, in the absence of lung-protective strategies, mechanical ventilation may contribute to ventilator-induced lung injury, driven by barotrauma or volutrauma resulting from elevated airway pressures or excessive tidal volumes (2). Furthermore, excessive oxygen administration has been implicated in the generation of reactive oxygen species (ROS), the development of absorption atelectasis, and impairment of alveolar gas exchange, all of which may exacerbate pulmonary injury (3). Notably, the LOco_2_ trial (4) in acute respiratory distress syndrome (ARDS) patients reported higher 90-day mortality in the low-oxygen target arm, prompting early study termination.

Conversely, a previous meta-analysis (5) evaluating different oxygenation targets in critically ill, mechanically ventilated patients suggested that liberal oxygen strategies may be associated with increased mortality. While providing valuable insights, that analysis was limited by variability in the definitions of conservative oxygen targets (COTs) and the inclusion of a heterogeneous population encompassing diverse critical illness phenotypes, potentially limiting the external validity of its conclusions. Since its publication, three additional randomized controlled trials (RCTs) (6–8) have reported no significant differences in outcomes between higher and lower oxygenation targets.

Given ongoing uncertainty regarding the risks and benefits of different oxygenation strategies, we conducted an updated systematic review and meta-analysis (OXY-BREATHES) comparing COT and liberal oxygen target (LOT) in mechanically ventilated ICU patients. This analysis focused on key clinical outcomes—including mortality, organ-support requirements, and relevant complications—to inform evidence-based oxygen therapy recommendations. To address limitations of prior work, we incorporated recent trials, standardized conservative oxygen definitions, and performed subgroup analyses.

## MATERIALS AND METHODS

This systematic review and meta-analysis were conducted in accordance with the Cochrane Collaboration Handbook for Systematic Review of Interventions and reported following the Preferred Reporting Items for Systematic Reviews and Meta-Analysis statement guidelines (9, 10).

### Eligibility Criteria

We included RCTs that directly compared COT vs. LOT in adult patients (≥ 18 yr old) admitted to an ICU and requiring mechanical ventilation. Studies were required to report at least one predefined clinical outcome of interest. COTs were defined as oxygen saturation (Spo_2_) between 88% and 94% or Pao_2_ less than or equal to 80 mm Hg. LOTs were defined more broadly, including Spo_2_ greater than 94%, Pao_2_ greater than or equal to 90 mm Hg, the use of Fio_2_ 1.0, or the absence of an upper oxygenation threshold.

We excluded studies defined conservative targets solely by Fio_2_ or lacked explicit Spo_2_/Pao_2_ criteria; pregnant patients; used a single-arm RCT design; were observational in nature or only published conference abstracts; included mixed populations with both ventilated and nonventilated patients; or were conducted exclusively in pediatric populations.

We also assessed cardiac and cerebral ischemic events and, where data allowed, performed subgroup analyses in patients with sepsis, acute brain injury (ABI), ARDS, and post-cardiac arrest to enhance clinical relevance. Patients were included in a subgroup if the original trial either focused exclusively on that population or reported outcomes separately. Subgroup data were extracted from published subgroup analyses or supplementary materials when available. We used the same random-effects model as in the main analysis, calculating relative risks for dichotomous outcomes and mean differences (MDs) for continuous outcomes. Given the limited data, we did not conduct formal interaction tests. These subgroup findings were considered hypothesis-generating rather than conclusive, with the underlying premise that conservative oxygenation may offer survival or secondary outcome benefits in these specific populations.

### Search Strategies and Data Extraction

A comprehensive search of major databases (PubMed, Embase, Scopus, and Cochrane Central) was performed from inception to June 2025. The full search strategies for each database are provided in **Appendix 1** (https://links.lww.com/CCM/H892). Reference lists of relevant systematic reviews and included studies were also manually screened for additional eligible trials. Study selection was conducted in two stages: titles and abstracts were independently screened by two reviewers (N.N., N.A.d.B.e.L.) to identify potentially eligible studies, followed by independent full-text review. Data extraction was performed independently and in duplicate by two reviewers (N.B.T., Y.Z.) using a standardized form, with forest plot data double-checked by two reviewers (H.D.T.L., V.N.D.N.). Discrepancies were resolved through discussion or, if needed, by a third reviewer (N.N.). This meta-analysis was prospectively registered on PROSPERO (CRD420251082611) on June 30, 2025.

Primary outcomes in the PROSPERO protocol were 30-day, 90-day, and ICU mortality, and ICU length of stay. In the final review, we retained ICU length of stay but prioritized 90-day mortality as the main mortality outcome, as it provided the longest follow-up, maximizing information and interpretability. Relative effects were generally consistent across time horizons. Although no additional outcomes were prespecified, we also analyzed clinically relevant secondary outcomes: complications (cardiac ischemia, cerebral ischemia, nosocomial pneumonia) and organ-support measures (ventilator-free days, vasopressor-free days, renal replacement therapy), addressing gaps arising from limited data from previous meta-analyses (5).

### Statistical Analysis

Two reviewers (N.B.T., Y.Z.) independently extracted baseline characteristics (**Table 1**) and outcome data. Dichotomous outcomes were pooled as risk ratios (RRs) with 95% CIs. Continuous outcomes were analyzed as MDs since all studies reported results in consistent units (d), which are more clinically interpretable than standardized MDs. A difference of greater than or equal to 1 day was considered the clinically important difference.

**TABLE 1.** Characteristics of All Included Studies

Study	Patient Population (Inclusion Criteria)	^a^COT, Range/*n*^a^	^a^LOT, Range/*n*	Primary Outcome	Age, Mean or Median COT/LOT	Pao_2_:Fio_2_ baseline, Median/Mean COT/LOT	^b^APACHE II or^c^SOFA, Median COT/LOT
Ghazaly et al (7) 2024 (RCT)	Adults ≥ 18 yr admitted to the surgical ICU with infection, a SOFA score ≥ 2 (Sepsis-3 criteria), and an anticipated need for mechanical ventilation for at least 72 hr	Spo_2_: 88–92%; Pao_2_: 60–75 mm Hg (*n* = 53)	Spo_2_: ≥ 96%; Pao_2_: 90–105 mm Hg (*n* = 53)	The stroke volume after 72 hr of oxygen therapy	48.0/52.6 (mean)	N/A	16/15 (APACHE II)
Asfar et al (11) 2017, HYPERS2S (RCT)	Septic shock and mechanically ventilated	Spo_2_: 88–95% (*n* = 217)	Fio_2_ of 1.0 for 24 hr after inclusion (*n* = 217)	All-cause mortality at 28 d post-randomization	66.3/67.8 (mean)	Mean: 228/220	10/10 (SOFA)
van der Wal et al (8) 2023, ICONIC (RCT^a^)	All patients ≥ 18 yr old expected to require mechanical ventilation for at least 24 hr were screened for eligibility	^a^Spo_2_: 91–94%;^a^Pao_2_: 55–80 mm Hg (*n* = 335)	Spo_2_: 96–100%; Pao_2_: 110–150 mm Hg (*n* = 329)	All-cause mortality at 28 d post-randomization	67/67 (median)	N/A	9/9 (SOFA)
Martin et al (12) 2021, TOXYC (RCT)	Eligible patients were mechanically ventilated adults (≥ 18 yr) within 24 hr of an unplanned ICU admission, and expected to require ventilation for at least 72 hr	Spo_2_: 88–92% (*n* = 17)	Spo_2_: ≥ 96% (*n* = 17)	The primary outcome was feasibility, defined by patient recruitment and withdrawal rates	66/66 (median)	Median: 265	11/11 (SOFA)
Martin et al (6) 2025, UK-ROX (RCT)	Patients (≥ 18 yr), enrolled within 12 hr of starting invasive mechanical ventilation after an unplanned ICU admission	Spo_2_: 88–92% (*n* = 8230)	No upper limit set Spo_2_ (*n* = 8204)	90-d mortality	60/60 (median)	Median: 200/198	16/16 (APACHE II)
Panwar et al (14) 2015 (RCT)	ICU patients ≥ 18 yr old were eligible if they had received invasive mechanical ventilation for < 24 hr and were expected by clinicians to require it for at least another 24 hr	Spo_2_: 88–92% (*n* = 52)	Spo_2_: ≥ 96% (*n* = 51)	The mean area under the curve for Spo_2_, arterial oxygen saturation, Pao_2_, and Fio_2_ on days 0–7	62.4/62.4 (mean)	Mean: 248/247	7.9/7.4 (SOFA [mean])
Barrot et al (4) 2020, LOco_2_ (RCT)	Patients were eligible if they had been intubated and on mechanical ventilation for < 12 hr due to acute respiratory distress syndrome (per the Berlin definition)	Spo_2_: 88–92%; Pao_2_: 55–70 mm Hg (*n* = 99)	Spo_2_: ≥ 96%; Pao_2_: 90–105 mm Hg (*n* = 102)	All-cause mortality at 28 d post-randomization	63.0/63.5 (mean)	Mean: 116.8/120.1	9.3/8.9 SOFA [mean])
Semler et al (13) 2022, PILOT (RCT)	All eligible adults (≥ 18 yr) in the medical ICU or in the emergency department with planned ICU admission were enrolled when they first received invasive mechanical ventilation	Spo_2_: 88–92% (low; *n* = 808)	Spo_2_: Spo_2_: ≥ 96% (high; *n* = 874)	Ventilator-free days	57/59 (median)	N/A	5/5 (SOFA)
Schmidt et al (15) 2022 (RCT)	Patients were comatose adults admitted after resuscitated cardiac arrest with sustained return of spontaneous circulation. All received targeted temperature management at 36°C, along with sedation and mechanical ventilation for at least 24 hr	Pao_2_: 68–75 mm Hg (*n* = 394)	Pao_2_: 98–105 mm Hg (*n* = 395)	Composite of death or discharge with severe disability/coma within 90 d post-randomization	62/63 (mean)	N/A	N/A

APACHE = Acute Physiology and Chronic Health Evaluation, COTs = conservative oxygen targets, LOTs = liberal oxygen targets, N/A = not applicable, RCT = randomized controlled trial, SOFA = Sequential Organ Failure Assessment, Spo_2_ = oxygen saturation.

a RCT, Spo_2_ (a noninvasive estimate of the Spo_2_ level of hemoglobin in the blood, measured by pulse oximetry, normal range at or above 95–100%), Pao_2_ (a direct measurement of the amount of oxygen dissolved in arterial blood, obtained via arterial blood gas analysis, normal range is 80–100 mm Hg on room air), COT, and LOT.

b The APACHE II score is a widely used severity-of-disease classification system for critically ill patients admitted to ICUs. The score ranges from 0 to 71, with higher scores indicating greater severity of illness and a higher risk of mortality.

c SOFA score is a clinical tool used to assess the degree of organ dysfunction in critically ill patients, especially those in the ICU. It evaluates six organ systems—respiratory, cardiovascular, liver, coagulation, renal, and neurologic—assigning each a score from 0 (normal function) to 4 (severe dysfunction). The total score ranges from 0 to 24, with higher scores indicating more severe organ failure and a higher risk of mortality. A SOFA score of 2 or more is considered indicative of significant organ dysfunction.

Heterogeneity was assessed using the *I*^2^ statistic and Cochrane Q test, with *I*^2^ greater than 25% and *p* value of less than 0.10 indicating substantial heterogeneity. All meta-analyses used a random-effects model (DerSimonian and Laird) to account for within- and between-study variability. Statistical analyses were conducted in Review Manager (RevMan), Version 5.4 (Cochrane Collaboration, Copenhagen, Denmark). Sensitivity analyses employed a leave-one-out approach, and subgroup analyses were performed when data allowed.

Another important consideration is that the PILOT trial used a cluster-randomized design. Although the original *NEJM* publication did not provide design-effect–adjusted estimates (such as those incorporating an intracluster correlation coefficient [ICC]), a subsequent reanalysis (16) by the trial’s primary investigators, published in *JAMA*, reported an ICC of 0.001, indicating minimal clustering. As the investigators concluded that no additional clustering adjustment was required, we therefore used the published effect estimates directly in our meta-analysis.

### Quality Assessment

Two independent reviewers (N.A.d.B.e.L., N.B.T.) assessed the risk of bias using the Cochrane Risk of Bias 2.0 (RoB 2) tool, across five domains: selection, performance, detection, attrition, and reporting bias. Each domain was rated as low, high, or unclear risk, with discrepancies resolved by consensus or a third reviewer (N.N.). Publication bias was evaluated using Egger’s test, and for outcomes with *p* value of less than 0.05, the trim-and-fill method was applied to adjust for potentially missing studies (analysis by D.D.).

The certainty of evidence was appraised using the Grading of Recommendations, Assessment, Development, and Evaluation (GRADE) (17) approach by (V.Q.T.H., D.D), considering risk of bias, imprecision, inconsistency, indirectness, and publication bias. Outcomes were rated as high, moderate, low, or very low certainty. All outcomes were evaluated to determine the overall strength and reliability of the OXY-BREATHES meta-analysis findings.

## RESULTS

### Study Selection and Baseline Characteristics

**Figure 1.**
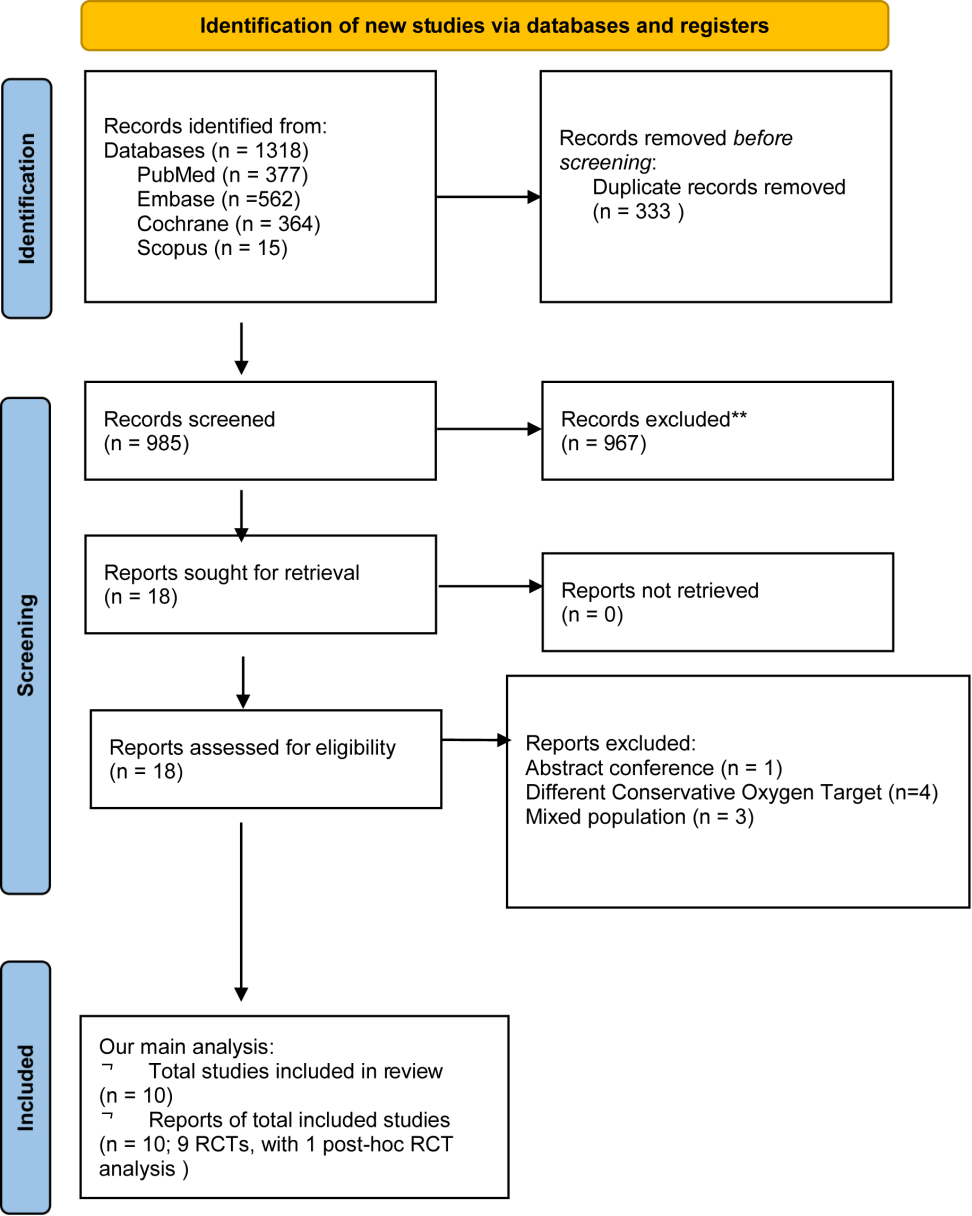
The Preferred Reporting Items for Systematic Reviews and Meta-Analyses flowchart (10) of study selection. RCT = randomized controlled trial.

The initial search identified 1318 records. After removing duplicates and screening titles and abstracts, 18 studies underwent full-text review. Eight trials were excluded: three for mixed ventilated and nonventilated populations (18–20), four for higher or nonstandard COT settings (21–24), and one conference abstract (25) (Supplementary Table 4, https://links.lww.com/CCM/H886). Ultimately, nine RCTs and one post hoc analysis of the PILOT trial (26) met inclusion criteria (**Fig. 1**), encompassing 20,447 mechanically ventilated ICU patients (10,205 assigned to COT and 10,242 to LOT). All trials included ICU patients receiving mechanical ventilation. Key subpopulations included septic shock (HYPERS2S [11]), sepsis (Ghazaly et al [8]), post-cardiac arrest (Schmidt et al [5]), and ARDS (LOco_2_ [4]). COTs were typically Spo_2_ 88–92%, except HYPERS2S (11) (88–95%), while liberal targets varied, including Spo_2_ greater than 94%, higher Fio_2_, or no upper Spo_2_ limit.

Across trials, males comprised greater than 55% of participants, and baseline illness severity, assessed by Sequential Organ Failure Assessment (SOFA) or Acute Physiology and Chronic Health Evaluation II scores, was balanced between groups. Six trials reported SOFA scores, with a median of greater than or equal to 5 in both COT and LOT groups (Table 1). Exclusion criteria varied (**Supplementary Table 5**, https://links.lww.com/CCM/H886), most commonly age younger than 18 years, pregnancy, and imminent risk of death. Trials excluding cardiac arrest patients included HYPERS2S (11), LOco_2_ (4), and TOXYC (12), while those excluding patients with brain injury or intracranial pathology were HYPERS2S (11), LOco_2_ (4), TOXYC (12), Schmidt et al (15), and ICONIC (8).

#### Mortality and Length of ICU Stay Outcomes

**Figure 2.**
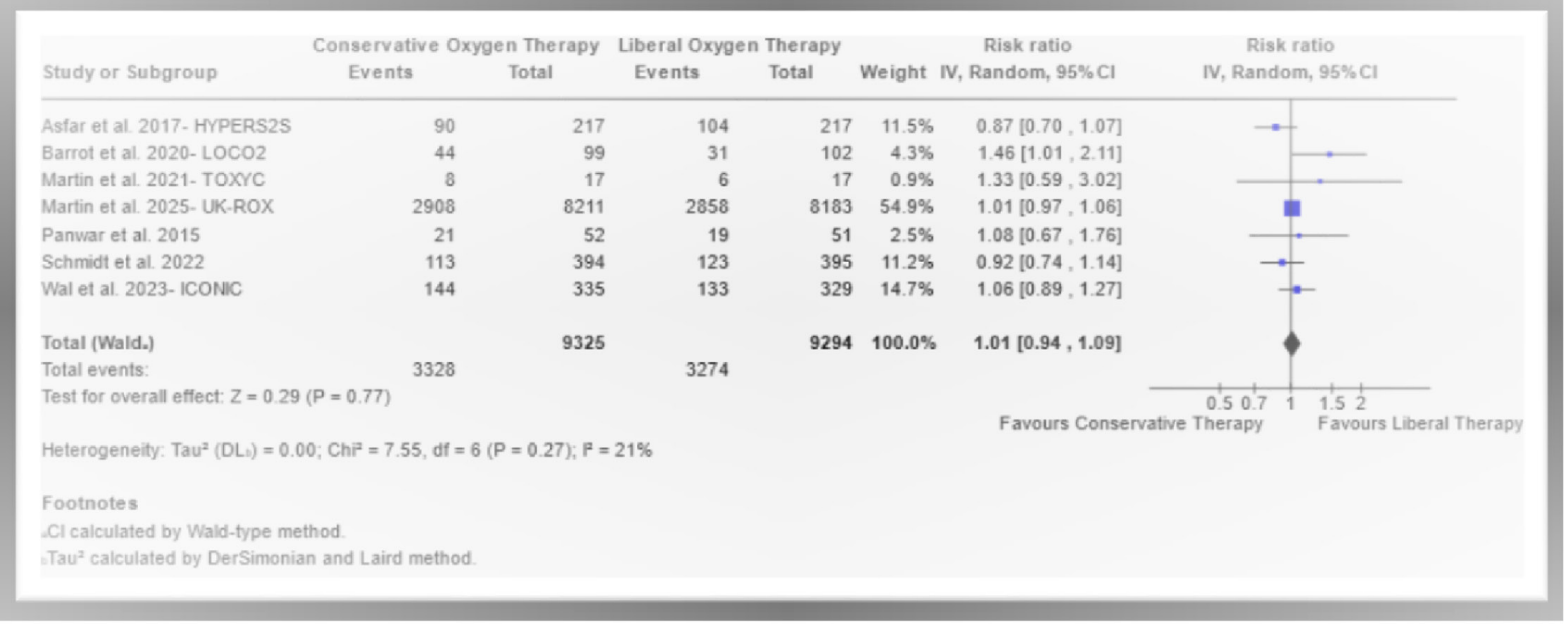
Ninety-day mortality outcomes between conservative oxygen targets and liberal oxygen targets. All analyses used a *p* < 0.05, with 95% CIs exclude null-value of 1 as statistical significance results. *df* = degrees of freedom, DL = DerSimonian-Laird.

**Figure 3.**
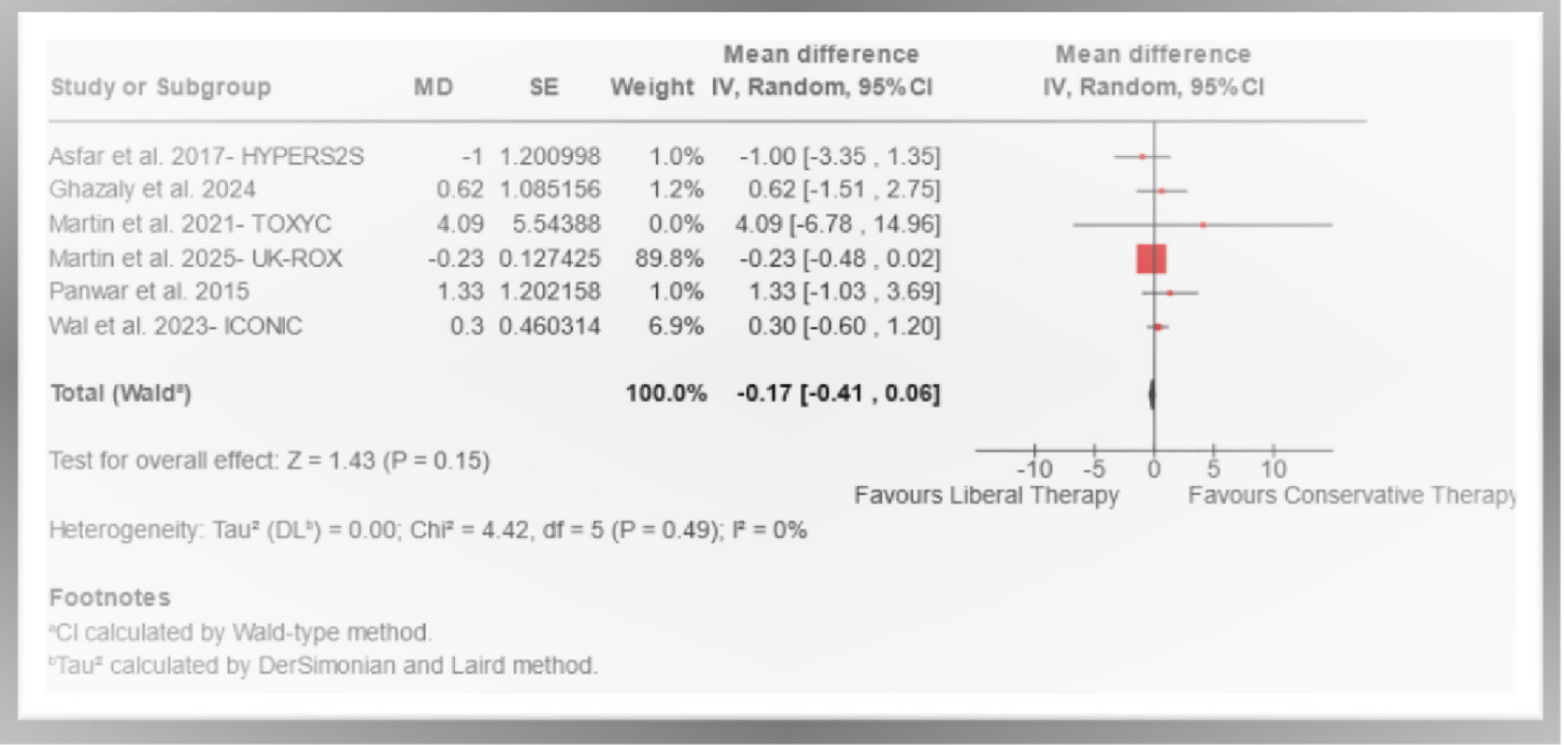
The mean difference (MD) in ICU length of stay (d) for patients treated with conservative oxygen target compared with liberal oxygen target therapy was analyzed. Statistical significance was defined as a *p* < 0.05 and 95% CIs that did not include the null value of 0. *df* = degrees of freedom, DL = DerSimonian-Laird.

In the OXY-BREATHES meta-analysis, there were no statistically significant differences between COT and LOT for the primary outcomes: 90-day mortality (RR, 1.01; 95% CI, 0.94–1.09; moderate certainty of evidence; **Fig. 2**) and ICU length of stay (MD, –0.17; 95% CI, –0.41 to 0.06; moderate certainty of evidence; **Fig. 3**).

#### Adverse Events and Organ-Support Outcomes

Adverse events, including cardiac ischemia, cerebral ischemia, and nosocomial pneumonia, showed no significant differences between conservative and liberal oxygenation: cardiac ischemia (RR, 0.85; 95% CI, 0.22–3.37; very low certainty of evidence; and **Supplementary Fig. 1**, https://links.lww.com/CCM/H886), cerebral ischemia (RR, 1.73; 95% CI, 0.58–5.13; very low certainty of evidence; and **Supplementary Fig. 2**, https://links.lww.com/CCM/H886), and nosocomial pneumonia (RR, 1.12; 95% CI, 0.80–1.56; low certainty of evidence; and **Supplementary Fig. 3**, https://links.lww.com/CCM/H886). Organ-support outcomes also did not differ significantly: ventilator-free days (MD, 0.37; 95% CI, –0.15 to 0.64; moderate certainty of evidence; and **Supplementary Fig. 4**, https://links.lww.com/CCM/H886), vasopressor-free days (MD, 0.35; 95% CI, –0.38 to 1.07; moderate certainty of evidence; and **Supplementary Fig. 5**, https://links.lww.com/CCM/H886), and renal replacement therapy (RR, 1.10; 95% CI, 0.87–1.38; low certainty of evidence; and **Supplementary Fig. 6**, https://links.lww.com/CCM/H886).

### Subgroup Analyses

#### Sepsis or Septic Shock

This subgroup analysis included four RCTs: HYPERS2S (11) (septic shock), Ghazaly et al (6) (sepsis), Shapiro et al (27) (sepsis/septic shock from the PILOT trial), and Martin et al (6) (sepsis/septic shock, 90-d mortality). Consistent with overall findings, conservative and liberal oxygen strategies showed no meaningful differences in mortality, renal replacement therapy, ICU length of stay, nosocomial pneumonia, or ventilator-free days (**Supplementary Figs. 7–11**, https://links.lww.com/CCM/H886). However, conservative targets were associated with more vasopressor-free days (MD, 2.00; 95% CI, 0.53–3.47; *p* = 0.008; *I*^2^ = 0%; and **Supplementary Fig. 12**, https://links.lww.com/CCM/H886). Definitions of sepsis and septic shock are provided in the **Supplement** (https://links.lww.com/CCM/H886).

#### Post-Cardiac Arrest

For post-cardiac arrest patients, three RCTs were initially identified (Supplementary Table 3, https://links.lww.com/CCM/H886): DeMasi et al (26) (post hoc PILOT analysis), which included both in-hospital and out-of-hospital arrests, and Jakkula et al (21) and Schmidt et al (15), limited to out-of-hospital arrests. Only two trials were ultimately included, as Jakkula et al (21) used a higher COT range (Pao_2_ 75–112.5 mm Hg) than our predefined criteria (Spo_2_ 88–94% or Pao_2_ ≤ 80 mm Hg). Both included trials reported mortality (DeMasi et al [26] for in-hospital and Schmidt et al [15] for 90-d mortality), but no secondary outcomes. COTs suggested a potential survival benefit in this subgroup (RR, 0.89; 95% CI, 0.79–1.00; *p* = 0.05; *I*^2^ = 0%; and **Supplementary Fig. 13**, https://links.lww.com/CCM/H886). However, with only 615 patients receiving COT, the sample size was limited. These findings should be interpreted cautiously, and further RCTs are needed to clarify the benefit of COT in this high-risk population.

#### ARDS and Acute Brain Injury

During our selection process, we identified one ARDS-specific trial (LOco_2_ [4]) and two trials focused on ABI, which were excluded (**Supplementary Table 2**, https://links.lww.com/CCM/H886): Lång et al (22) (traumatic brain injury) and Young et al (23) (mixed ABI cohort). Lång et al (22) assessed Fio_2_ alone without specifying Spo_2_ or Pao_2_ targets, while Young et al (23) applied a COT range less than 97%, above our predefined threshold. Although UK-ROX (6) reported an ABI subgroup, the limited data prevented separate analyses for ARDS or ABI.

### Quality Assessment

#### Risk of Bias

Using the RoB 2 tool (28), most trials in OXY-BREATHES were rated as having “some concerns” for overall risk of bias. Although objective endpoints like mortality reduce the impact of performance or detection bias, all trials; except Ghazaly et al (6), raised concerns regarding deviations from intended interventions, mainly due to open-label conservative oxygen arms (**Supplementary Table 1**, https://links.lww.com/CCM/H886). Egger’s test indicated no publication bias (*p* > 0.05).

#### GRADE Assessment

None of our outcomes were rated as high certainty, as most studies (except Ghazaly et al [7]) were at risk of deviations from intended interventions. Two outcomes, cerebral ischemia and cardiac ischemia, were rated very low due to imprecision and risk of bias from open-label designs (**Supplementary GRADE Table**, https://links.lww.com/CCM/H886).

#### Sensitivity Analysis

We also examined the impact of methodological variations in Asfar et al (11), which applied a shorter intervention duration (< 24 hr) and a slightly higher Spo_2_ range (88–95%) than our predefined conservative target (88–94%). Excluding this trial again produced results consistent with the main analysis of primary outcomes, with no significant differences between conservative and liberal oxygenation for ICU length of stay (*p* = 0.18; **Supplementary Fig. 14*A***, https://links.lww.com/CCM/H886) or 90-day mortality (*p* = 0.44; **Supplementary Fig. 14*A***, https://links.lww.com/CCM/H886), confirming the robustness and stability of our main analysis.

## DISCUSSION

This meta-analysis (OXY-BREATHES), including nine RCTs and 20,447 mechanically ventilated ICU patients, found no significant differences in key outcomes (mortality, ICU length of stay, or organ support-free days) between conservative (Spo_2_ 88–94%) and liberal (> 94%) oxygen strategies. Notably, our meta-analysis systematically evaluate subgroups of critically ill, mechanically ventilated patients. In patients with sepsis or septic shock, conservative oxygenation was associated with more vasopressor-free days, while mortality and adverse event rates remained similar.

In sepsis, the observed increase in vasopressor-free days with conservative oxygenation may be biologically plausible. Hyperoxia can exacerbate endothelial injury, platelet activation, and ROS production, all of which contribute to the pathogenesis of disseminated intravascular coagulation (29). Limiting arterial oxygen exposure may thus help preserve hemodynamic stability and reduce vasopressor dependence.

Compared with prior meta-analyses, several methodological refinements improved the specificity and clinical applicability of findings. First, we replaced two earlier trials that included both ventilated and nonventilated patients (HOT-ICU [19] and OXYGEN-ICU [20]) with three more recent RCTs that exclusively enrolled mechanically ventilated patients (ICONIC 2023 [8], Ghazaly et al [7], UK-ROX 2025 [6]). Third, we conducted subgroup analyses (sepsis and post-cardiac arrest) in patients with specific conditions, in whom oxygen therapy may exert differential effects. Fourth, we defined clearly COTs (Spo_2_: 88–94% or Pao_2_ below 80 mm Hg) to address inconsistency of the intervention definitions noted in earlier meta-analyses.

The rationale for setting COTs at Spo_2_ 88–94% is as follows. First, most of the trials included in our meta-analysis defined conservative oxygenation within the 88–92% range, with the exception of Asfar et al (11), which used 88–95%. Second, this range is both practical and potentially safe for critically ill, mechanically ventilated ICU patients. It remains above the steep portion of the oxyhemoglobin dissociation curve, avoiding dangerously low-oxygen levels, while also staying below the plateau where small increases in Fio_2_ could lead to excessive oxygenation (30). This ensures adequate tissue oxygen delivery while minimizing the risk of hyperoxia-related harm (31–33).

Within our post-cardiac subgroup analysis, our hypothesis for such observed potential survival benefit may stem from reduced reperfusion injury. Excessive oxygen delivery during reperfusion is known to amplify ROS formation, thereby worsening ischemia-reperfusion injury and systemic inflammation (34). This phenomenon is analogous to myocardial stunning observed after acute myocardial infarction, wherein transient cardiac dysfunction follows reperfusion (35).

A separate analysis for the ABI subgroup was not feasible due to the lack of eligible trials. Subgroup data from UK-ROX (6) (odds ratio, 0.86; 95% CI, 0.56–1.34) suggest no meaningful difference between COT and LOT strategies. This may reflect the predominant influence of cerebral perfusion and blood pressure on outcomes, rather than oxygen levels (36). Additionally, inflammation and impaired autoregulation during acute injury may further limit the benefit of supplemental oxygen in this population.

In the ARDS subgroup, we observed a signal of higher 90-day mortality, reported only in the LOco_2_ trial, despite no differences in short-term outcomes (30-d or ICU mortality; Fig. 2). This suggests that COT may exert delayed adverse effects. As Barrot et al (4) proposed, reduced oxygen radical formation under COT might initially attenuate lung injury; however, in ARDS, where baseline oxygenation is already compromised, further restriction may worsen hypoxemia over time. Prolonged hypoxemia could contribute to intestinal ischemia, impaired immunity, and bacterial translocation, potentially increasing late mortality (37, 38). These findings underscore the need for further studies to clarify the safety of COTs in ARDS.

Our analysis has several limitations. First, trauma-focused trials were excluded, and data from TRAUMOX2 (18) were inconclusive and included nonventilated patients. Second, only one trial specifically enrolled ARDS patients (4), and UK-ROX (6) provided limited ABI subgroup data, preventing dedicated analyses for these populations. Third, sample sizes were small in the cardiac arrest and sepsis subgroups, along with sepsis and septic shock were combined due to reporting constraints, potentially diluting condition-specific effects. Finally, despite standardizing conservative oxygen definitions, residual heterogeneity persisted across critical illness subtypes, along with variability in liberal oxygen ranges.

COTs of Spo_2_ 88–94% in mechanically ventilated ICU patients should be applied in a tailored, condition-specific manner, as oxygen needs vary across clinical subgroups. Emerging evidence suggests that conservative oxygenation may confer benefit in patients with sepsis and following cardiac arrest, whereas caution is warranted in those with ARDS. Although a substantial body of literature has examined oxygenation targets, most trials have applied broad inclusion criteria irrespective of the underlying acute condition precipitating ICU admission, thereby introducing significant heterogeneity. Nevertheless, two ongoing large-scale RCTs—OPTI-OXYGEN (39) and MEGA-ROX (40–42)—are expected to provide more definitive evidence on conservative oxygen strategies, particularly in patients with sepsis and the underexplored ABI population. Notably, MEGA-ROX, with its exceptionally large sample size of approximately 40,000 patients, includes predefined subgroups for hypoxic-ischemic encephalopathy (HIE), sepsis, and non-HIE neurologic conditions. Furthermore, within the post-cardiac arrest population, the LOGICAL substudy of MEGA-ROX (43), which has already completed recruitment, will specifically address the effects of conservative vs. liberal oxygenation in cardiac arrest survivors.

## CONCLUSIONS

In this OXY-BREATHES meta-analysis of nine RCTs including 20,447 mechanically ventilated ICU patients, COTs (Spo_2_ 88–94% or Pao_2_ < 80 mm Hg) were comparable to liberal targets (Spo_2_ ≥ 94% or Pao_2_ ≥ 90 mm Hg) for mortality, ICU length of stay, organ support-free days, nosocomial pneumonia, and ischemic events. In subgroup analyses, conservative targets were associated with more vasopressor-free days in sepsis and suggested a potential survival benefit in post-cardiac arrest patients. These findings highlight the need for future condition-specific RCTs to define optimal oxygenation strategies tailored to individual critically ill subgroups.

## ACKNOWLEDGMENTS

We gratefully acknowledge the unwavering support and encouragement of our families throughout the course of this study. We also thank Dr. Rhanderson Cardoso of the Meta-analysis Academy for his expert guidance on meta-analytic methodology. Last, we acknowledge the Faculty of Medicine, University of Debrecen, for providing foundational training in critical care medicine, which enabled us undertake this research.

## Supplementary Material


