# Supplementary material for: Conservative Oxygen Targets in Mechanically Ventilated Patients (OXY-BREATHES): A Systematic Review and Meta-Analysis of Randomized Controlled Trials
Source: Crit Care Med. 2026 Feb 9;54(5):1225–36. doi: 10.1097/CCM.0000000000007031 (PMC13134654; doi:10.1097/CCM.0000000000007031)
Supplement: Supplementary file 1 [file ccm-54-1225-s001.docx]

**APPENDIX 1- SEARCH STRATEGY**

## We systematically searched PubMed, Embase, Scopus and Cochrane Central Register of Controlled Trials from inception to June 2025, with the following terms: ‘Oxygen Targets’, ‘Oxygenation Strategies’, ‘ICU’, ‘Liberal Oxygen’, ‘Conservative Oxygen’, ‘Hypoxia’, ‘Critical Care’, ‘Mechanical Ventilation’. Our search strategy was built for PubMed based on the above terms and their synonyms as follows:

## ("liberal oxygen" OR "conservative oxygen" OR "oxygen targets" OR "oxygenation strategies" OR "hyperoxia" OR "hypoxia" OR "low oxygen" OR "high oxygen") AND ("respiration, artificial" OR "mechanical ventilation" OR "positive-pressure respiration" OR "ventilated patients") AND ("intensive care units" OR "critical care" OR ICU OR "intensive care" OR "critically ill") AND (randomized controlled trial[pt] OR controlled clinical trial[pt] OR randomized[tiab] OR placebo[tiab] OR "clinical trials as topic"[Mesh])

Specifically for Embase, we used the following high-sensitive search strategy: ('liberal oxygen':ti,ab OR 'conservative oxygen':ti,ab OR hyperoxia:ti,ab OR hypoxia:ti,ab OR 'low oxygen':ti,ab OR 'high oxygen':ti,ab) AND ('respiration artificial':ti,ab OR 'mechanical ventilation':ti,ab OR 'positive-pressure respiration':ti,ab OR 'ventilated patients':ti,ab) AND ('intensive care units':ti,ab OR 'critical care':ti,ab OR icu:ti,ab OR 'intensive care':ti,ab OR 'critically ill':ti,ab) AND (random*:ti,ab,kw OR 'clinical trial*':ti,ab,kw OR 'health care quality'/exp OR 'health care quality')
