# Supplementary material for: Conservative Oxygen Targets in Mechanically Ventilated Patients (OXY-BREATHES): A Systematic Review and Meta-Analysis of Randomized Controlled Trials
Source: Crit Care Med. 2026 Feb 9;54(5):1225–36. doi: 10.1097/CCM.0000000000007031 (PMC13134654; doi:10.1097/CCM.0000000000007031)
Supplement: Supplementary file 2 [file ccm-54-1225-s002.docx]

**OXY-BREATHES Study**

**Term definitions:**

**Sepsis:** A condition marked by confirmed infection together with signs of organ dysfunction, impaired perfusion, or low blood pressure. It is typically diagnosed when infection is present along with at least two SIRS criteria (such as fever, hypothermia, or abnormal white blood cell count) and evidence of organ compromise, including low blood oxygen, elevated creatinine, or clotting abnormalities. Other signs can include elevated lactate levels and delayed capillary refill.

**Septic shock:** A severe form of sepsis where hypotension persists despite adequate fluid administration, accompanied by signs of inadequate tissue perfusion such as elevated lactate, reduced urine output, or altered mental status. Notably, patients on vasopressor support may have masked hypotension.

**Pooled analysis of all included studies**

**Conservative Oxygen Targets (COT)**: target SpO2 goal of **88-94%** considered as conservative therapy, or Po2 goal of 70 mmHg or less.

**Liberal Oxygen Targets (LOT)**: Target Sp02 goal **≥95%**, or Po2 goal above 90 mmHg.

**Supplementary Figure 1. Cardiac Ischemia:**

**Conservative oxygen targets (COT) versus Liberal oxygen targets (LOT).**


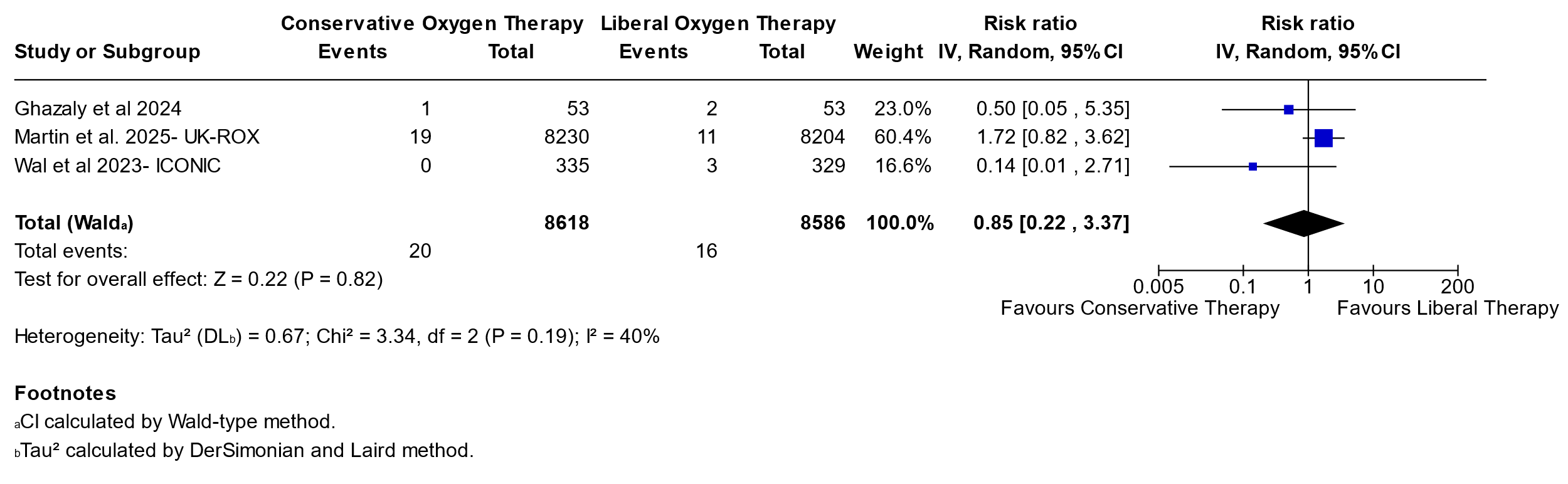


Figure 4. There were no noticeable differences in cardiac ischemia events for patients treated with COT, compared to LOT therapy (RR 0.85; 95% CI 0.22-3.37; p=0.82).

**Eggers (P = 0.2858), intercept = -8.3165 (95% CI: -59.2168 – 42.5838)**

**Supplementary Figure 2. Cerebral Ischemia:**

**Conservative oxygen targets (COT) versus Liberal oxygen targets (LOT).**


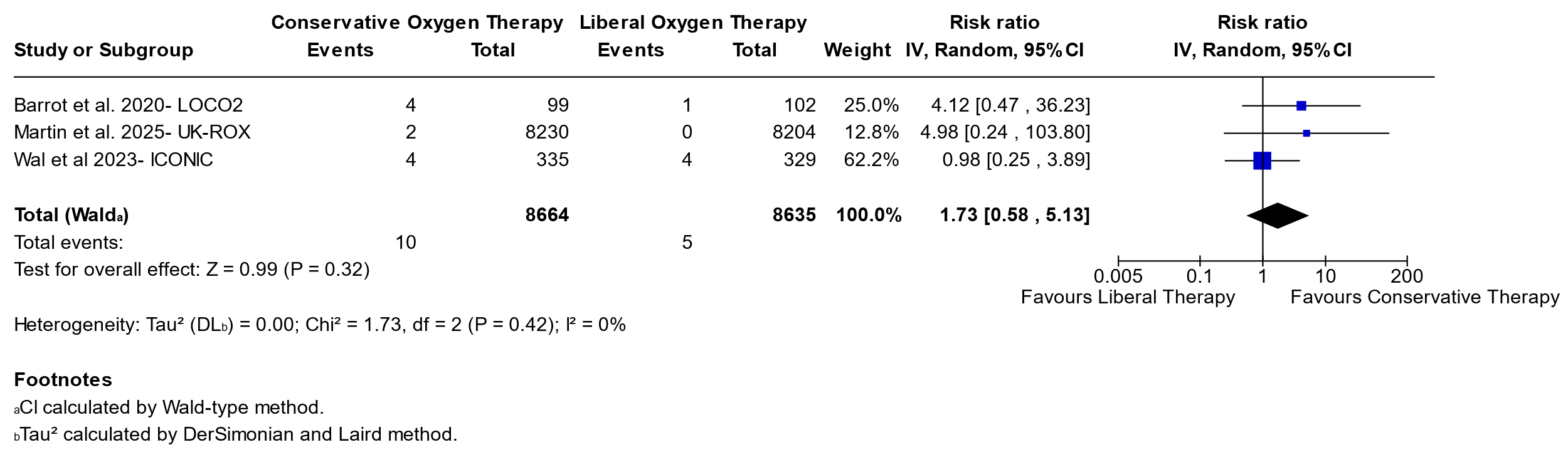


Figure 5. There were no statistically noticeable differences in the incidence of cerebral ischemia between patients treated with Conservative therapy (COT) and those receiving liberal therapy (LOT) (RR 1.73; 95% CI: 0.58–5.13; p = 0.32).

**Eggers (P = 0.7868), intercept = -0.6612 (95% CI: -24.8012 – 23.4787**

**Supplementary Figure 3. Nosocomial Pneumonia:**

**Conservative oxygen targets (COT) versus Liberal oxygen targets (LOT).**


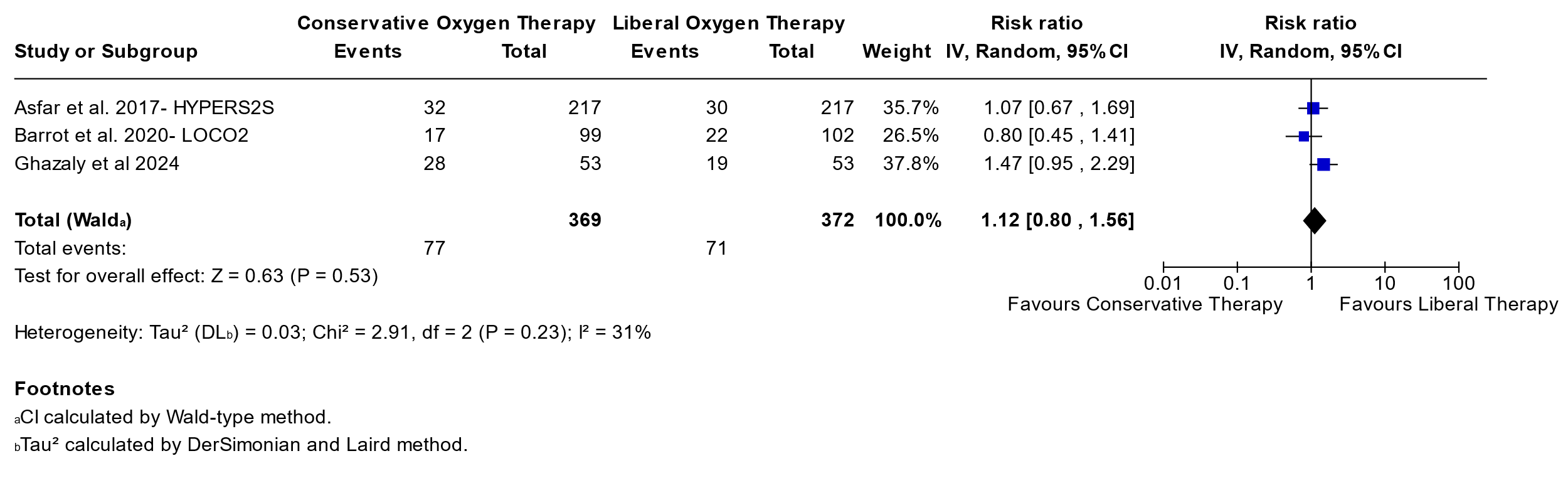


Figure 6. There were minimal differences in nosocomial pneumonia risk between patients treated with COT and those received LOT therapy (RR 1.12; 95% CI 0.80-1.56; p=0.53). Events ratio was 1.09 between COT and LOT.

**Eggers (P = 0.2858), intercept = -8.3165 (95% CI: -59.2168 – 42.5838)**

**Supplementary Figure 4 . Ventilator-free day:**

**Conservative oxygen targets (COT) versus Liberal oxygen targets (LOT).**


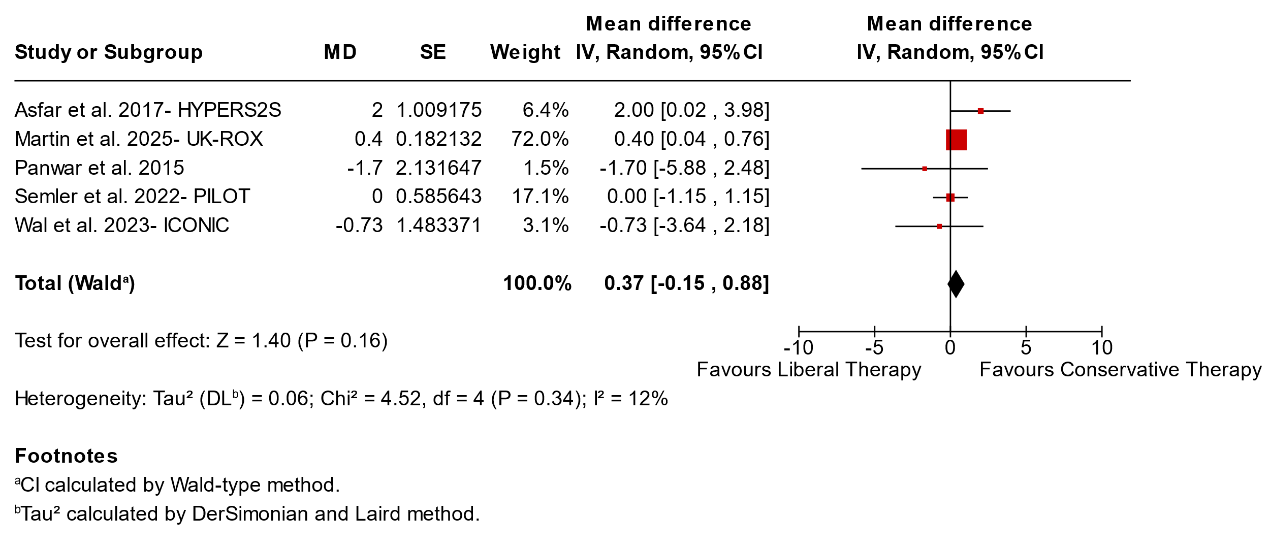


Figure 7. COT was comparable to LOT in term of mean ventilator-free days (MD 0.37; 95% CI -0.15-0.64; p=0.88).

**Eggers: P = 0.5211, intercept = 0.1604 (CI: -0.2434 - 0.5641)**

**Supplementary Figure 5. Vasopressor-free days:**

**Conservative oxygen targets (COT) versus Liberal oxygen targets (LOT).**


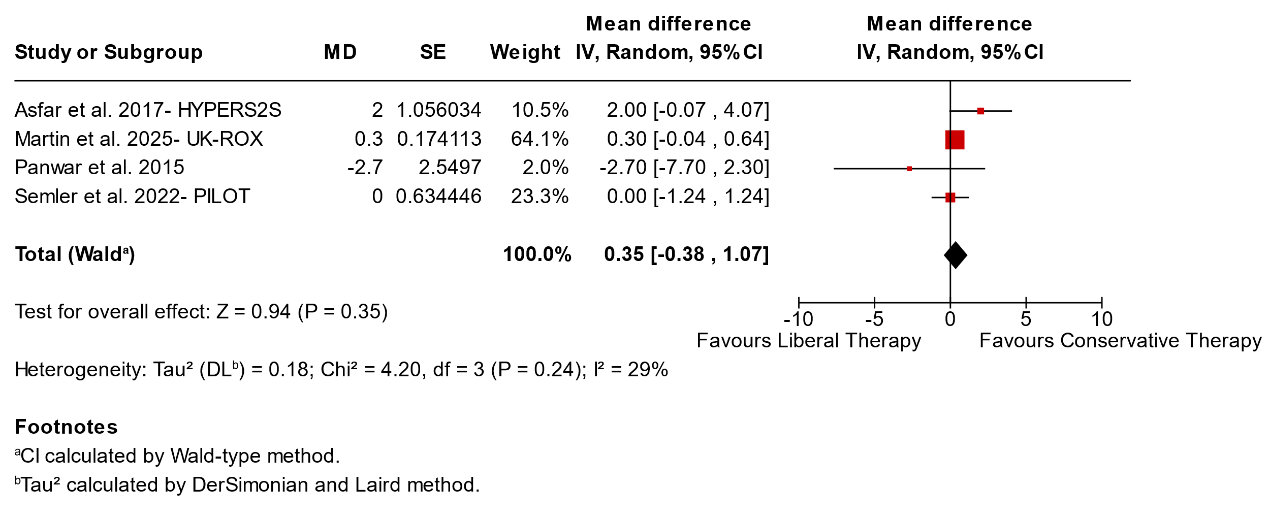


Figure 8. COT was comparable to LOT in term of mean vasopressor-free days (MD 0.35; 95% CI -0.38-1.07; p=0.35).

**Eggers (P = 0.4793), interecept = 0.1674 (CI: -0.3102 – 0.6451)**

**Supplementary Figure 6. Renal replacement therapy:**

**Conservative oxygen targets (COT) versus Liberal oxygen targets (LOT).**


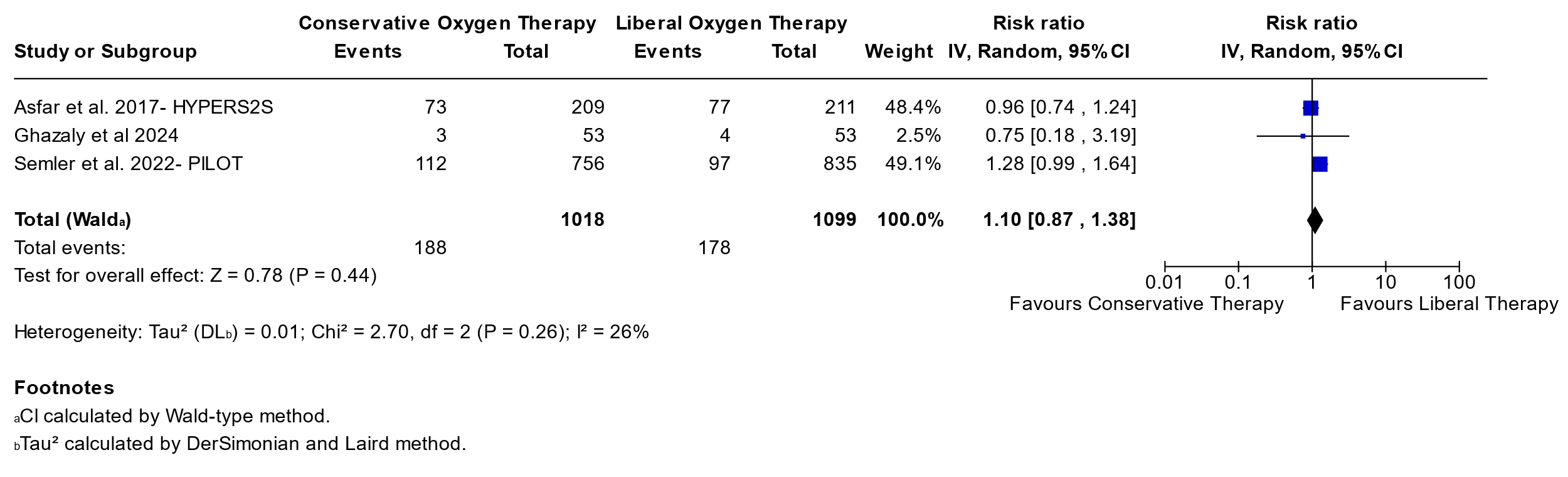


Figure 9. There were no noticeable differences in renal replacement therapy for patients treated with COT, compared to LOT therapy (RR 1.10; 95% CI 0.87-1.38; p=0.44).

**Eggers (P = 0.7868), intercept = -0.6612 (95% CI: -24.8012 – 23.4787)**

**Sepsis or Septic Shock subgroup**

The following studies are included in this analysis: HYPERS2S focused on septic shock subgroup, Ghazaly et al 2024 focused on sepsis, Shapiro et al 2025 focused on sepsis or septic shock subgroup of main trial PILOT, and finally Martin et al 2025 provided only 90-day mortality of sepsis or septic shock subgroup. Overall, the results of subgroup are aligned with the main pooled analysis. There were no noticeable differences between COT and LOT regarding Mortality, renal replacement therapy, length of ICU stay,nosocomial pneumonia risks and finally ventilator-free days. **However, the conservative therapy COT might be associated with more vasopressor-free day compared to liberal therapy LOT, which may favor this strategy in sepsis subgroup.**

**Supplementary Figure 7. 90-day Mortality:**

**Conservative oxygen targets (COT) versus Liberal oxygen targets (LOT).**

*
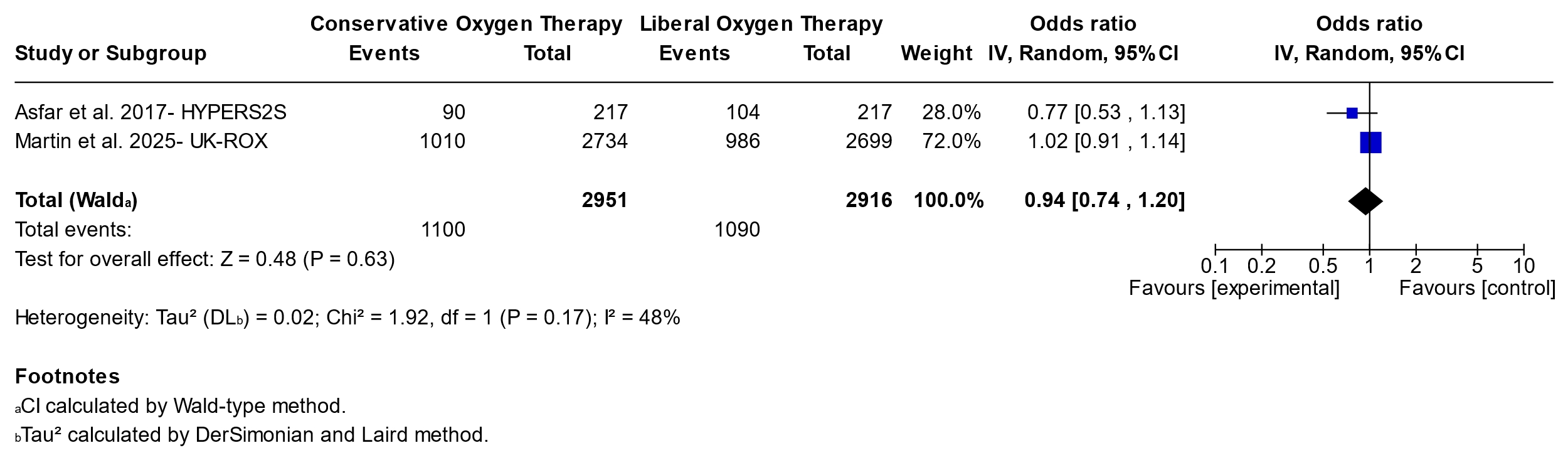
*

**Supplementary Figure 8 . Renal replacement therapy:**

**Conservative oxygen targets (COT) versus Liberal oxygen targets (LOT).**

**
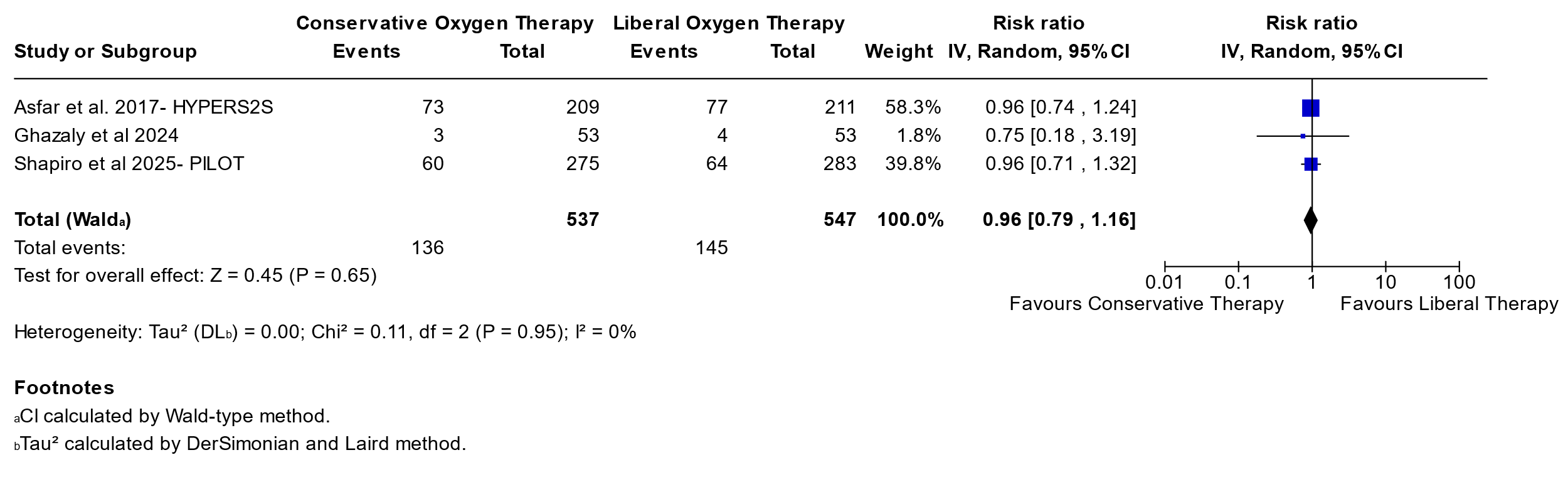
**

**Supplementary Figure 9. Length of stay in ICU:**

**Conservative oxygen targets (COT) versus Liberal oxygen targets (LOT).**


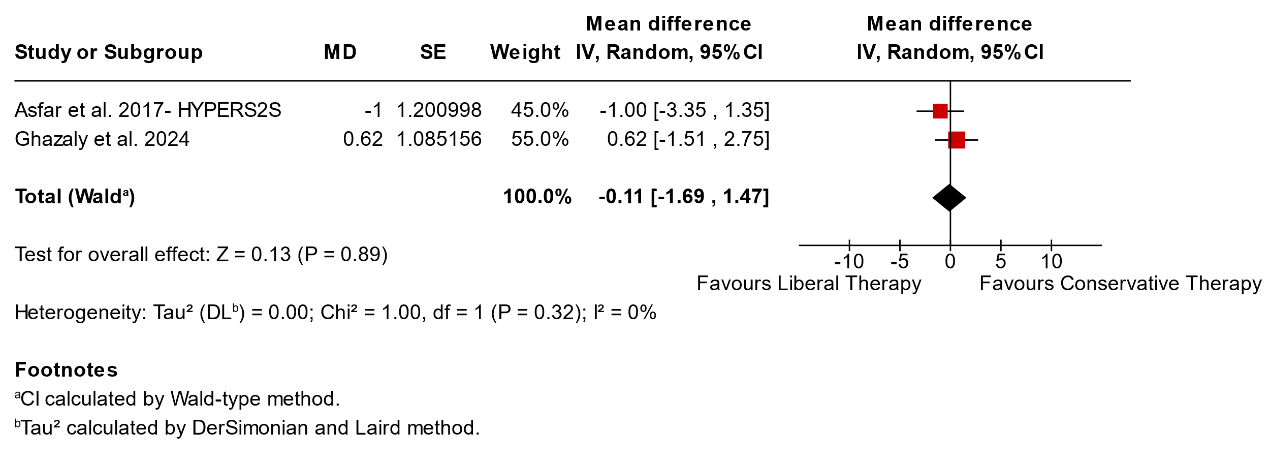


**Supplementary Figure 10. Nosocomial Pneumonia:**

**Conservative oxygen targets (COT) versus Liberal oxygen targets (LOT).**

**
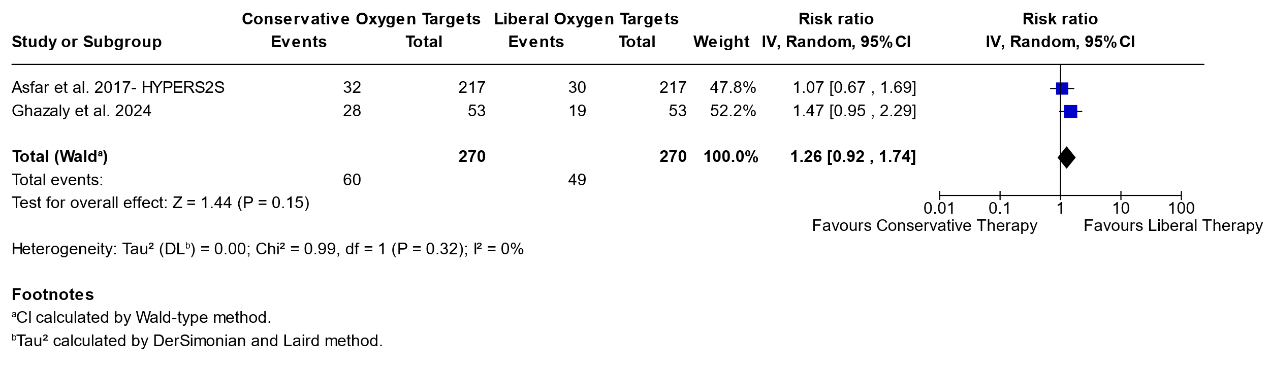
**

**Supplementary Figure 11. Ventilator-free days:**

**Conservative oxygen targets (COT) versus Liberal oxygen targets (LOT).**

**
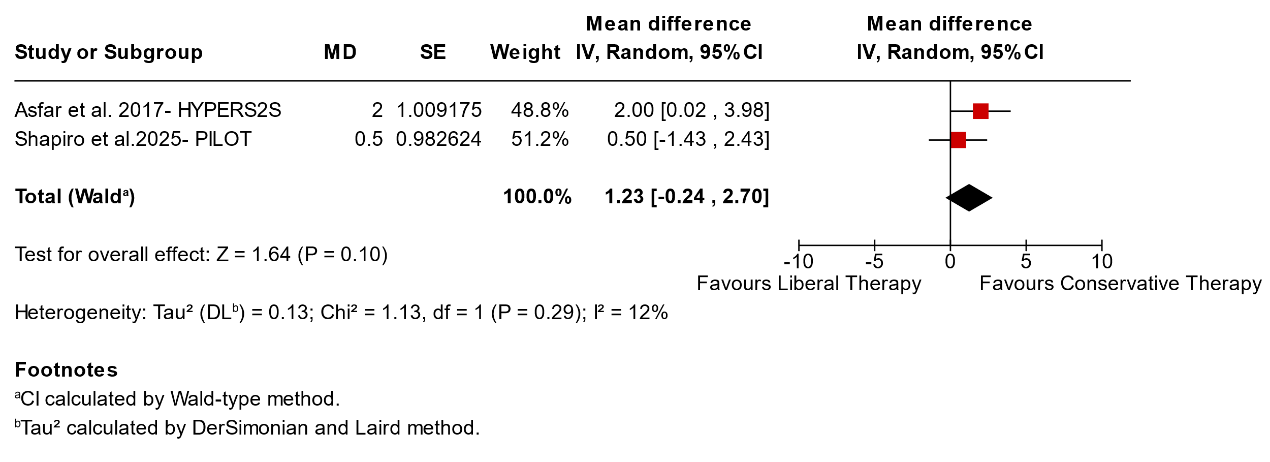
**

**Supplementary Figure 12. Vasopressor-free days:**

**Conservative oxygen targets (COT) versus Liberal oxygen targets (LOT).**

**
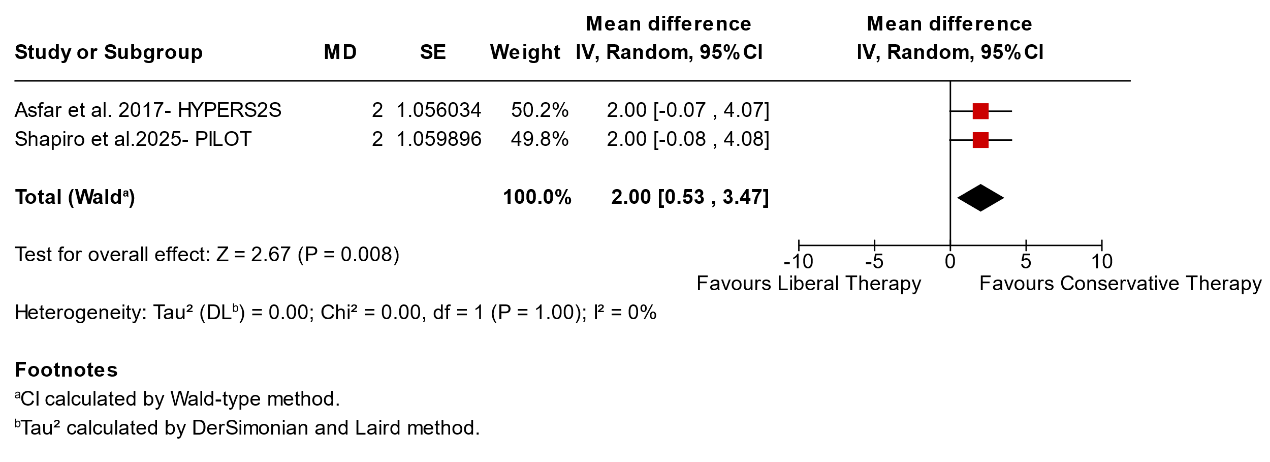
**

**Post-Cardiac Arrest subgroup**

Interestingly, **COT appeared to be associated with improved survival outcomes compared to LOT in patients who had suffered cardiac arrest, regardless locations.**

**Supplementary Figure 13. Cardiac Arrest, without Jakkula et al. 2018**

**Conservative oxygen targets (COT) versus Liberal oxygen targets (LOT).**

**
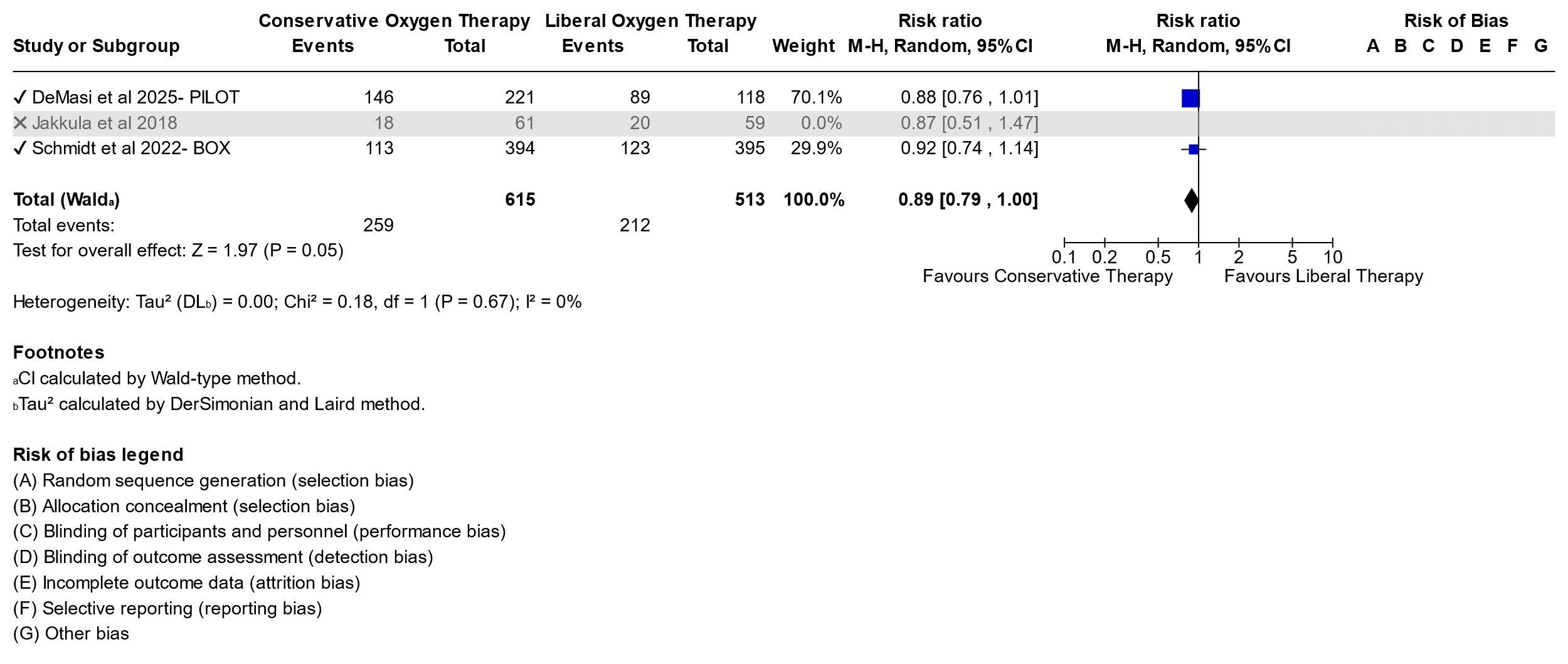
**

**Supplementary Figure 14. Sensitivity analysis: Asfar et al 2017 exclusion**

1. **ICU length of stay**


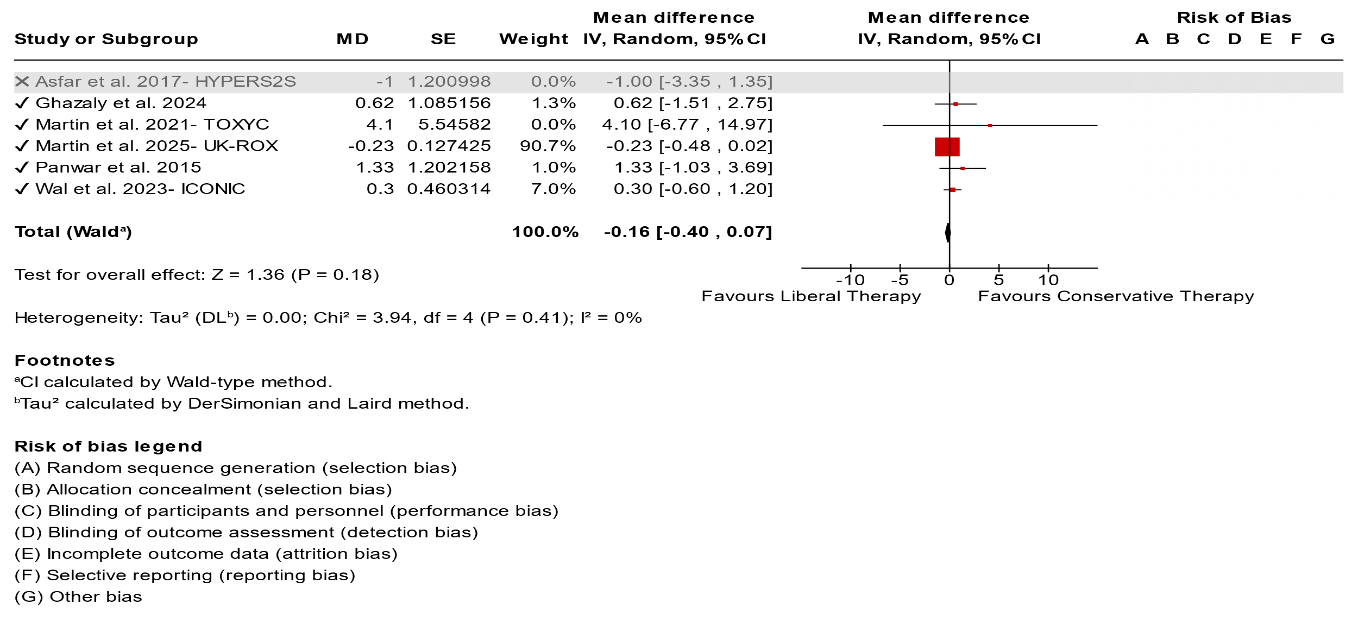


**B. 90-day Mortality**


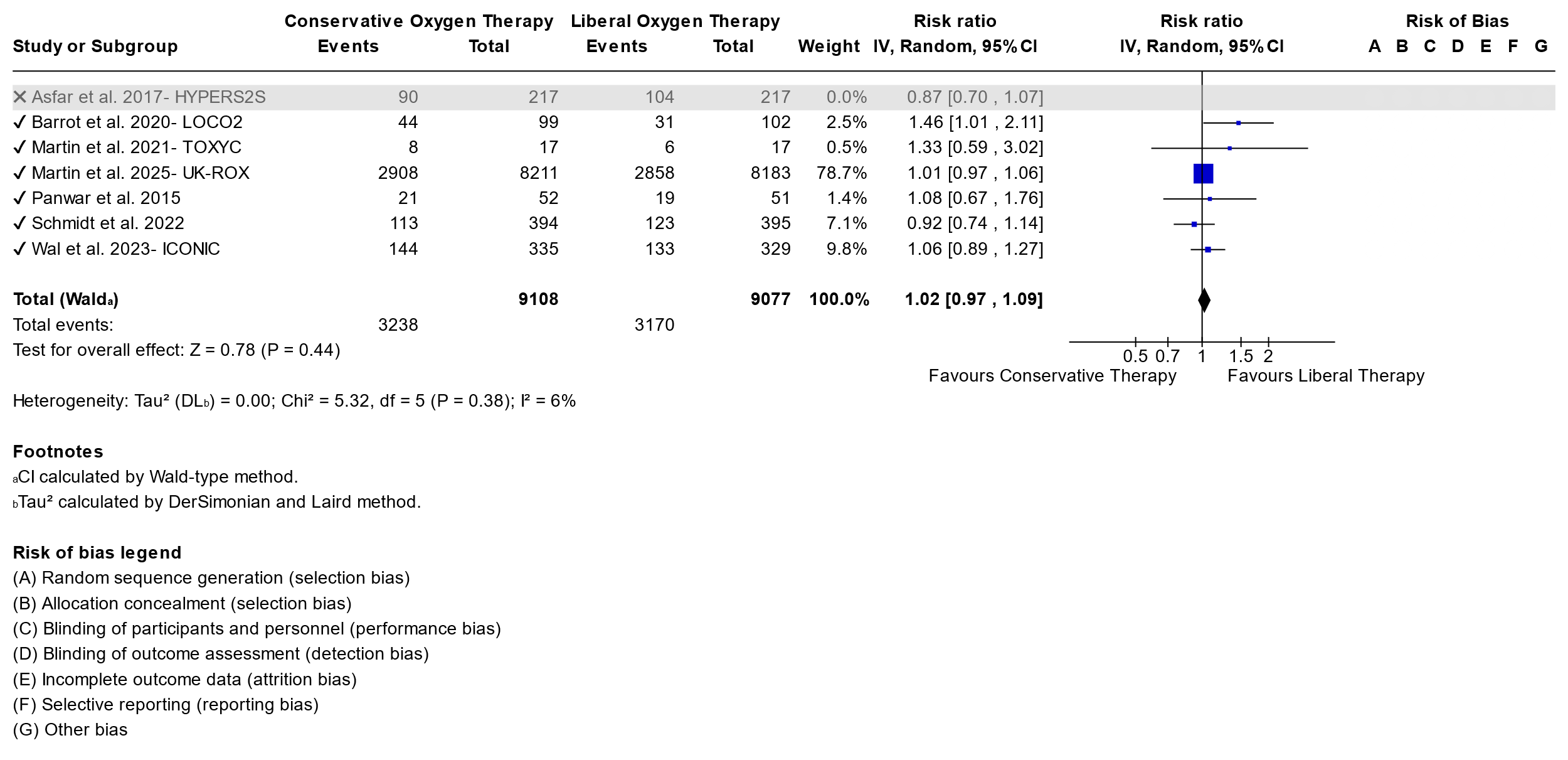


**Supplementary Table 1. Risk of bias summary for randomized studies (RoB 2)**

| **Study** | **Bias from randomization process** | **Bias due to deviations from intended interventions** | **Bias due to missing outcome data** | **Bias in measurement of the outcomes** | **Bias in selection of the reported result** | **Overall risk of bias** |
| --- | --- | --- | --- | --- | --- | --- |
| Ghazaly et al. 2024 | Low | Low | Low | Low | Low | Low |
| Asfer et al. 2017  HYPERS2S | Low | Some concerns | Low | Low | Low | Some Concerns |
| Wal et al. 2023- ICONIC | Low | Some concerns | Low | Low | Low | Some  Concerns |
| Martin et al. 2021  TOXYC | Low | Some concerns | Low | Low | Low | Some  Concerns |
| Martin et al. 2025  UK-ROX | Low | Some concerns | Low | Low | Low | Some  Concerns |
| Panwar et al 2015 | Low | Some concerns | Low | Low | Low | Some Concerns |
| Semler et al 2022  PILOT | Low | Some concerns | Low | Low | Low | Some  Concerns |
| Barrot et al 2020  LOCO2 | Low | Some concerns | Low | Low | Low | Some Concerns |
| Schmidt et al. 2022 | Low | Some concerns | Low | Low | Low | Some  Concerns |

**Table 2** summarizes the risk of bias assessments for the included studies. The majority of studies were determined to have a low-to-some-concern overall risk of bias, with no studies classified as being at high risk. All trials—except for Ghazaly et al. (2024)—had some concerns regarding bias due to deviations from intended interventions, primarily because of their open-label design in conservative oxygen targets.

**Supplementary Table 2.**

**Characteristics of all excluded studies focused on Acute Brain Injury**

|  | **Lang et al. 2018**  **(N=65)** | | **Young et al. 2022**  **(N=217)** | |
| --- | --- | --- | --- | --- |
| **Characteristics** | COT^%^  (n=27) | LOT*  (n=38) | COT  (n=112) | LOT  (n=105) |
| Study design | RCT^@^ | | RCT | |
| COT, definition | FiO2 = 0.4 | | FiO₂ was minimized to 0.2 whenever feasible, with SpO₂ kept below 97%, and an upper alarm limit set at 97% to avoid hyperoxia. | |
| LOT, definition | FiO2 = 0.7 | | No specific measures were implemented to limit FiO₂ or SpO₂, except that setting upper alarm limits for SpO₂ was not allowed, and the use of FiO₂ below 0.3 during invasive ventilation was discouraged. | |
| ABI^+^ types | TBI | | TBI, SAH, ICH | |
| Age, years (mean) | 43 | 45 | 51.8 | 51.8 |
| ^#^APACHE II, mean | 22 | 23 | 21.1 | 21.2 |
| Male, % | 85.18 | 81.57 | 59.8 | 59 |
| TBI, n | 27 | 38 | 45 | 43 |

% COT: Conservative oxygen targets , LOT*: Liberal oxygen targets

@RCT: Randomized Controlled Trials

+ABI: Acute Brain Injury including Traumatic Brain Injury (TBI), Subarachnoid Hemorrhage (SAH), and Intracranial Hemorrhage (ICH)

#The APACHE II score (Acute Physiology and Chronic Health Evaluation II) is a widely used severity-of-disease classification system for critically ill patients admitted to intensive care units (ICUs). The score ranges from 0 to 71, with higher scores indicating greater severity of illness and a higher risk of mortality

**Supplementary Table 3.**

**Characteristics of studies focused on Cardiac Arrest**

|  | **DeMasi et al. 2025**  **(N=65)**  **INCLUDED** | | **Jakkula et al. 2018**  **(N=217)**  **EXCLUDED** | | **Schmidt et al. 2022**  **(N=788)**  **INCLUDED** | |
| --- | --- | --- | --- | --- | --- | --- |
| **Characteristics** | ^%^COT  (n=27) | *LOT  (n=38) | COT  (n=112) | LOT  (n=105) | COT  (n=394) | LOT  (n=395) |
| Study design | RCT | | RCT | | RCT | |
| COT, definition | Lower spO2:  88%-92%  Intermediate spO2:  92-96% | | PaO2:75-112.5 mmHg | | PaO2: 68-75 mmHg | |
| LOT, definition | Higher spO2:  96%-100% | | PaO2: 150-187.5 mmHg | | PaO2: 98-105 mmHg | |
| Location of cardiac arrest | Mixed  (both in-and-out of hospital) | | Out-of-hospital | | Out-of-hospital | |
| Age, years (mean) | 59 | 60 | 61 | 58 | 62 | 63 |
| ^#^GCS score at admission, median | 3 | 4 | 3 | 3 | N/A | N/A |
| Male, % | 56.8 | 55.9 | 80 | 83 | 82.5 | 79.0 |
| Immediate coronary angiography, % | N/A | N/A | 58 | 47 | 92.1 | 90.9 |
| Time to ROSC^&^, median, minutes | 10 | 10 | 22 | 19 | 21 | 21 |

% COT: Conservative oxygen targets , LOT*: Liberal oxygen targets

@RCT: Randomized Controlled Trials

&ROSC: **ROSC** stands for **Return of Spontaneous Circulation**.It refers to the **resumption of a sustained, perfusing heartbeat and effective blood circulation** after cardiac arrest, typically following cardiopulmonary resuscitation (CPR) or advanced cardiac life support (ACLS)

#GCS score (Glasgow Coma Scale) ranges from 3 to 15 and assesses three components: eye opening, verbal response, and motor response. A lower score indicates a more severe brain injury.

**Supplementary Table 4. All Excluded Studies**

| **Study** | **Reason of exclusion,**  **based on selection criteria of Method part** |
| --- | --- |
| ***Jakkula et al. 2018 [32]*** | COT with PaO2 was set above 75 mmHg, compare to our predefined COT range for PaO2 (below 80 mmHg) |
| ***Lang et al. 2018 [33]*** | COT and LOT were both defined by solely FiO2, without further SpO2 or PaO2 range |
| ***Young et al. 2022 [34]*** | Even though the trial used SpO2 for COT and LOT, the upper limit for COT was up till 97%, 3% higher than our COT range (88%-92%) |
| ***HOT-ICU [29]*** | Even though trial broadly included patients admitted with different acute illnesses with pneumonia dominantly, the mixed ventilated and non-ventilated populations would introduce further heterogeneity in our analysis |
| ***OXYGEN-ICU [30]*** | the mixed ventilated and non-ventilated populations would introduce further heterogeneity in our analysis. Further, the conservative oxygen targets (94%-98%) were higher compared to our COT |
| ***ICU-ROX [40]*** | the ICU-ROX trial[38] was excluded because its conservative arm used an SpO₂ range of 90–97% with the same lower limit as the liberal arm, making it incompatible with our predefined conservative target (88–94%). This exclusion ensured consistency in the conservative oxygen definition and improved the validity of effect estimates. |
| ***TRAUMOX 2 [22]*** | Even though trial focused on patients suffered from trauma, the mixed ventilated and non-ventilated populations would introduce further heterogeneity in our analysis |
| ***Barrot et al 2018 [41]*** | No outcomes of interests as trials focused on pro-inflammatory cytokines, sRAGE and organ dysfunctions, as well as results were published in solely as an abstract in conference |

| **Supplementary Table 5.** | **Exclusion criteria of each study** |
| --- | --- |
| ***Asfar et al 2017***  ***HYPERS2S (RCT) [12]*** | Exclusion criteria included severe hypoxaemia (PaO₂:FiO₂ < 100 mm Hg with PEEP ≥ 5 cm H₂O), plasma sodium <130 or >145 mmol/L**, intracranial hypertension, admission for cardiac arrest,** overt cardiac failure, legal guardianship, lack of affiliation with the French healthcare system, pregnancy, recent participation in another biomedical or interventional study with mortality as the primary endpoint, or the investigator’s decision not to resuscitate. |
| ***Barrot et al. 2020***  ***LOCO2 (RCT) [15]*** | The main exclusion criteria were long-term home oxygen therapy or noninvasive ventilation, and ICU admission due to **cardiac arrest, traumatic brain injury, or cranial hypertension.** |
| ***Ghazaly et al 2024 (RCT) [6]*** | Exclusion criteria were age <18 years, pregnancy, acute respiratory distress syndrome, chronic obstructive pulmonary disease, idiopathic pulmonary fibrosis, cardiovascular or chronic kidney disease, and refusal to participate. |
| ***Martin et al. 2021***  ***TOXYC (RCT) [13]*** | Exclusion criteria included ICU admission following surgery (elective or unplanned), expected death within 24 hours, pregnancy, **post-cardiac arrest,** chronic lung disease with baseline SpO₂ in the intervention range (88–92%), **post-trauma (including traumatic brain injury**), sickle cell trait or disease, ongoing significant hemorrhage or severe anemia, severe peripheral vascular or pulmonary disease, other conditions contraindicating mild hypoxaemia, and participation in another intervention. |
| ***Martin et al. 2025***  ***UK-ROX (RCT) [5]*** | Exclusion criteria included prior randomization to UK-ROX within 90 days, receipt of extracorporeal membrane oxygenation, or if the treating clinician deemed the intervention clinically indicated or contraindicated. |
| ***Panwar et al. 2015***  ***(RCT) [16]*** | Exclusion criteria were known pregnancy, imminent risk of death, or lack of clinician equipoise for trial enrollment. |
| ***Semler et al 2022***  ***PILOT (RCT) [14]*** | Patients were excluded if pregnant or incarcerated. |
| ***Schmidt et al. 2022* (RCT)[36]** | Exclusion criteria were: consciousness (obeying verbal commands); females of childbearing potential without a negative HCG test; in-hospital cardiac arrest; out-of-hospital cardiac arrest of non-cardiac origin (e.g., trauma, major arterial rupture, drowning, suffocation, hanging); known bleeding diathesis (excluding medically induced coagulopathy such as warfarin, NOACs, or clopidogrel); suspected or confirmed acute intracranial bleeding or stroke; unwitnessed asystole; limitations of therapy or DNR order; conditions precluding 180-day survival; pre-arrest Cerebral Performance Category 4; >4 hours from ROSC to screening; systolic blood pressure <80 mmHg despite support; or admission temperature <30 °C. |
| ***Wal et al. 2023***  ***ICONIC (RCT*) [7]*** | The main exclusion criteria were a decision to withhold life-sustaining treatment, ARDS with PaO₂/FiO₂ < 150 mm Hg, acute decompensation of COPD, severe low cardiac output shock (cardiac index < 2 L/min/m²), venoarterial extracorporeal membrane oxygenation, conditions requiring hyperoxygenation, severe non-reversible anemia (hemoglobin < 4.0 mmol/L), and uncontrollable intracranial hypertension. |

**Supplementary Table 6. PRISMA checklist.**

| **Section and Topic** | **Item #** | **Checklist item** | **Page where item is reported** |
| --- | --- | --- | --- |
| **TITLE** | | |  |
| Title | 1 | Identify the report as a systematic review. | 1 |
| **ABSTRACT** | | |  |
| Abstract | 2 | See the PRISMA 2020 for Abstracts checklist. | 3-4 |
| **INTRODUCTION** | | |  |
| Rationale | 3 | Describe the rationale for the review in the context of existing knowledge. | 5 |
| Objectives | 4 | Provide an explicit statement of the objective(s) or question(s) the review addresses. | 5-6 |
| **METHODS** | | |  |
| Eligibility criteria | 5 | Specify the inclusion and exclusion criteria for the review and how studies were grouped for the syntheses. | 7 |
| Information sources | 6 | Specify all databases, registers, websites, organisations, reference lists and other sources searched or consulted to identify studies. Specify the date when each source was last searched or consulted. | 8 |
| Search strategy | 7 | Present the full search strategies for all databases, registers and websites, including any filters and limits used. | 8 |
| Selection process | 8 | Specify the methods used to decide whether a study met the inclusion criteria of the review, including how many reviewers screened each record and each report retrieved, whether they worked independently, and if applicable, details of automation tools used in the process. | 8 |
| Data collection process | 9 | Specify the methods used to collect data from reports, including how many reviewers collected data from each report, whether they worked independently, any processes for obtaining or confirming data from study investigators, and if applicable, details of automation tools used in the process. | 8 |
| Data items | 10a | List and define all outcomes for which data were sought. Specify whether all results that were compatible with each outcome domain in each study were sought (e.g. for all measures, time points, analyses), and if not, the methods used to decide which results to collect. | 9 |
|  | 10b | List and define all other variables for which data were sought (e.g. participant and intervention characteristics, funding sources). Describe any assumptions made about any missing or unclear information. | N/A |
| Study risk of bias assessment | 11 | Specify the methods used to assess risk of bias in the included studies, including details of the tool(s) used, how many reviewers assessed each study and whether they worked independently, and if applicable, details of automation tools used in the process. | 10 |
| Effect measures | 12 | Specify for each outcome the effect measure(s) (e.g. risk ratio, mean difference) used in the synthesis or presentation of results. | 9 |
| Synthesis methods | 13a | Describe the processes used to decide which studies were eligible for each synthesis (e.g. tabulating the study intervention characteristics and comparing against the planned groups for each synthesis (item #5)). | N/A |
|  | 13b | Describe any methods required to prepare the data for presentation or synthesis, such as handling of missing summary statistics, or data conversions. | N/A |
|  | 13c | Describe any methods used to tabulate or visually display results of individual studies and syntheses. | N/A |
|  | 13d | Describe any methods used to synthesize results and provide a rationale for the choice(s). If meta-analysis was performed, describe the model(s), method(s) to identify the presence and extent of statistical heterogeneity, and software package(s) used. | 10 |
|  | 13e | Describe any methods used to explore possible causes of heterogeneity among study results (e.g. subgroup analysis, meta-regression). | 10 |
|  | 13f | Describe any sensitivity analyses conducted to assess robustness of the synthesized results. | 10 |
| Reporting bias assessment | 14 | Describe any methods used to assess risk of bias due to missing results in a synthesis (arising from reporting biases). | 10 |
| Certainty assessment | 15 | Describe any methods used to assess certainty (or confidence) in the body of evidence for an outcome. | 10 |
| **RESULTS** | | |  |
| Study selection | 16a | Describe the results of the search and selection process, from the number of records identified in the search to the number of studies included in the review, ideally using a flow diagram. | 13 |
|  | 16b | Cite studies that might appear to meet the inclusion criteria, but which were excluded, and explain why they were excluded. | 13-14 |
| Study characteristics | 17 | Cite each included study and present its characteristics. | 15 |
| Risk of bias in studies | 18 | Present assessments of risk of bias for each included study. | 21 |
| Results of individual studies | 19 | For all outcomes, present, for each study: (a) summary statistics for each group (where appropriate) and (b) an effect estimate and its precision (e.g. confidence/credible interval), ideally using structured tables or plots. | 17-21 |
| Results of syntheses | 20a | For each synthesis, briefly summarise the characteristics and risk of bias among contributing studies. | N/A |
|  | 20b | Present results of all statistical syntheses conducted. If meta-analysis was done, present for each the summary estimate and its precision (e.g. confidence/credible interval) and measures of statistical heterogeneity. If comparing groups, describe the direction of the effect. | 13-16 |
|  | 20c | Present results of all investigations of possible causes of heterogeneity among study results. | 23 |
|  | 20d | Present results of all sensitivity analyses conducted to assess the robustness of the synthesized results. | 23 |
| Reporting biases | 21 | Present assessments of risk of bias due to missing results (arising from reporting biases) for each synthesis assessed. | 21 |
| Certainty of evidence | 22 | Present assessments of certainty (or confidence) in the body of evidence for each outcome assessed. | 22 |
| **DISCUSSION** | | |  |
| Discussion | 23a | Provide a general interpretation of the results in the context of other evidence. | 26 |
|  | 23b | Discuss any limitations of the evidence included in the review. | 28 |
|  | 23c | Discuss any limitations of the review processes used. | 28 |
|  | 23d | Discuss implications of the results for practice, policy, and future research. | 26 |
| **OTHER INFORMATION** | | |  |
| Registration and protocol | 24a | Provide registration information for the review, including register name and registration number, or state that the review was not registered. | 4 |
|  | 24b | Indicate where the review protocol can be accessed, or state that a protocol was not prepared. | N/A |
|  | 24c | Describe and explain any amendments to information provided at registration or in the protocol. | N/A |
| Support | 25 | Describe sources of financial or non-financial support for the review, and the role of the funders or sponsors in the review. | 30 |
| Competing interests | 26 | Declare any competing interests of review authors. | 2 |
| Availability of data, code and other materials | 27 | Report which of the following are publicly available and where they can be found: template data collection forms; data extracted from included studies; data used for all analyses; analytic code; any other materials used in the review. | 30 |

*From:*  Page MJ, McKenzie JE, Bossuyt PM, Boutron I, Hoffmann TC, Mulrow CD, et al. The PRISMA 2020 statement: an updated guideline for reporting systematic reviews. BMJ 2021;372:n71. doi: 10.1136/bmj.n71. This work is licensed under CC BY 4.0. To view a copy of this license, visit <https://creativecommons.org/licenses/by/4.0/>

**Supplementary GRADE TABLE:**

| **Conservative Oxygen Targets (COT) compared to Liberal Oxygen Targets (LOT). for Mechanically Ventilated Patients** | | | | | |
| --- | --- | --- | --- | --- | --- |
| **Patient or population:** Mechanically Ventilated Patients  **Setting: ICU**  **Intervention:** Conservative Oxygen Targets (LOT)  **Comparison:** Liberal Oxygen Targets (HOT). | | | | | |
| **Outcomes** | **№ of participants (studies) Follow-up** | **Certainty of the evidence (GRADE)** | **Relative effect (95% CI)** | **Anticipated absolute effects** | |
|  |  |  |  | **Risk with Conservative Oxygen Targets (COT).** | **Risk difference with Liberal Oxygen Targets (LOT)** |
| 90-day Mortality (90-day Mortality) assessed with: Risk Ratio | 17830 (6 RCTs) | ⨁⨁⨁◯ Moderate^a,d^ | **RR 1.03** (0.93 to 1.13) | 354 per 1,000 | **11 more per 1,000** (25 fewer to 46 more) |
| Length of stay in ICU (Length of stay in ICU) assessed with: Standard Mean Difference | (6 RCTs) | ⨁⨁⨁◯ Moderate^d^ | - | - | SMD **0.02 SD lower** (0.06 higher to 0.01 higher) |
| Ventilator-free day (Ventilator-free day) assessed with: Standard Mean Difference | (6 RCTs) | ⨁⨁⨁◯ Moderate^d^ | - | - | SMD **0.04 SD higher** (0.01 lower to 0.09 higher) |
| Nosocomial Pneumonia assessed with: Risk Ratio | 741 (3 RCTs) | ⨁⨁◯◯ Low^b,d^ | **RR 1.12** (0.80 to 1.56) | 191 per 1,000 | **23 more per 1,000** (38 fewer to 107 more) |
| Vasopressor-free days (Vasopressor-free days) assessed with: Standard Mean Difference | (4 RCTs) | ⨁⨁⨁◯ Moderate^d^ | - | - | SMD **0.03 SD higher** (0.03 lower to 0.1 higher) |
| Renal replacement therapy (Renal replacement therapy) assessed with: Risk Ratio | 2117 (3 RCTs) | ⨁⨁◯◯ Low^b,d^ | **RR 1.10** (0.87 to 1.38) | 162 per 1,000 | **16 more per 1,000** (21 fewer to 62 more) |
| Cardiac Ischemia (Cardiac Ischemia) assessed with: Risk Ratio | 17204 (3 RCTs) | ⨁◯◯◯ Very Low^b,d^* | **RR 0.85** (0.22 to 3.37) | 2 per 1,000 | **0 fewer per 1,000** (1 fewer to 4 more) |
| Cerebral Ischemia (Cerebral Ischemia) assessed with: Risk Ratio | 17299 (3 RCTs) | ⨁◯◯◯ Very Low^b,d^* | **RR 1.73** (0.58 to 5.13) | 1 per 1,000 | **0 fewer per 1,000** (0 fewer to 2 more) |
| ***The risk in the intervention group** (and its 95% confidence interval) is based on the assumed risk in the comparison group and the **relative effect** of the intervention (and its 95% CI).  **CI:** confidence interval; **RR:** risk ratio; **SMD:** standardised mean difference | | | | | |
| **GRADE Working Group grades of evidence** **High certainty:** we are very confident that the true effect lies close to that of the estimate of the effect. **Moderate certainty:** we are moderately confident in the effect estimate: the true effect is likely to be close to the estimate of the effect, but there is a possibility that it is substantially different. **Low certainty:** our confidence in the effect estimate is limited: the true effect may be substantially different from the estimate of the effect. **Very low certainty:** we have very little confidence in the effect estimate: the true effect is likely to be substantially different from the estimate of effect. | | | | | |

#### Explanations

a. **Inconsistency** was judged by forest plot inspection, I², and Chi² rather than I² cut-offs

b. **Imprecision** was rated down when 95% CIs crossed narrow clinical thresholds of importance.

Downgrading for imprecision may be considered if the 95% CI of an RR crosses below 0.75 or above 1.25. For dichotomous outcomes, a two-level downgrade may be warranted when the ratio of the upper to lower CI boundary exceeds 2.5 for ORs or 3 for RRs, reflecting a wide interval (*).

c. **Publication bias** was assessed using Egger’s test; if p < 0.05, certainty of evidence was downgraded for risk of publication bias.

d. **Risk of bias**: Most studies (except Ghazaly et al. 2024) downgraded for deviations from intended interventions due to open-label design.
